# A Review on Modified Montmorillonite-Based Catalysts for Biofuel and Recycled Carbon Fuel Production

**DOI:** 10.3390/molecules31020339

**Published:** 2026-01-19

**Authors:** Ouahiba Madjeda Mecelti, Denys Grekov, Sary Awad

**Affiliations:** 1 IMT Atlantique, GEPEA, UMR CNRS 6144, 4 Rue Alfred Kastler, 44307 Nantes, France; ouahiba-madjeda.mecelti@imt-atlantique.fr (O.M.M.); denys.grekov@imt-atlantique.fr (D.G.); 2 Agence de l’environnement et de la Maîtrise de l’Energie, 20, Avenue du Grésillé-BP 90406, Cedex 01, 49004 Angers, France

**Keywords:** montmorillonite, biomass, plastics, pyrolysis, marine biofuels

## Abstract

The maritime transport sector’s reliance on fossil-based fuels remains a major contributor to global greenhouse gas emissions, underscoring the urgent need for sustainable alternatives such as marine biofuels. Thermochemical pyrolysis of biomass and plastic waste represents a promising route for producing renewable and recycled marine fuel feedstocks. This review provides an integrated analysis of the full production and upgrading chain, encompassing pyrolysis of lignocellulosic biomass and polymer-derived resources, catalytic upgrading, and qualitative evaluation of product distribution and yield trends. Particular emphasis is placed on montmorillonite-based catalysts as naturally abundant, low-cost, and environmentally benign alternatives to conventional zeolites. The review systematically examines the influence of key montmorillonite modification strategies, including acid activation, pillaring, and ion-exchanged, on acidity, textural properties, and catalytic performance in catalytic cracking and hydrodeoxygenation processes. The analysis shows that catalyst modification strongly governs the yield, selectivity, and reproducibility of biofuels. By adopting this integrated perspective, the review extends beyond existing works focused on isolated upgrading steps or zeolitic catalysts. Key research gaps are identified, particularly regarding long-term catalyst stability, deep deoxygenation of real bio-oils, and compliance with marine fuel standards.

## 1. Introduction

### 1.1. Climate Change Mitigation in Maritime Transport

Climate change remains one of the most urgent global challenges of the 21st century. Despite increasing awareness and international commitments, greenhouse gas (GHG) emissions and global temperatures continue to rise [1]. The COVID-19 pandemic in 2020 temporarily disrupted this trend, leading to a 5.8% reduction in global CO_2_ emissions due to reduced industrial and transport activity. However, this drop was short-lived. By 2021, emissions had surged again, reaching 33 gigatons (Gt), highlighting the deep dependence of modern economies on fossil fuels [2]. To limit global warming to 1.5 °C, the international community has set a target of achieving net-zero carbon emissions by 2050. Meeting this goal requires a fundamental transformation of how energy is produced, distributed, and consumed. While electricity from renewable sources is expected to play a major role, not all sectors can easily be electrified. In areas like shipping, aviation, and heavy industry, low-emission fuels and alternative technologies are urgently needed. In the maritime sector, which was responsible for about 800 MtCO_2_ in 2020 [2], emission reductions have been pursued mainly through operational strategies, including slower travel speeds, larger vessels, and efficiency monitoring. Nevertheless, these measures remain insufficient, as combustion inefficiencies and particle formation persist. Therefore, increasing attention is directed toward cleaner fuels and advanced carbon-reduction technologies [3], essential to achieving the ambitious climate targets set by the International Maritime Organization.

### 1.2. Alternative Fuels in Maritime Transport

**Figure 1:**
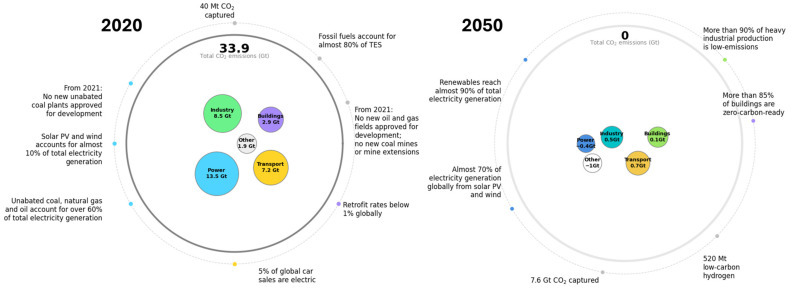
Roadmap to Net Zero Emissions by 2050 according to the IEA; Reproduced from ref. [2]. Copyright © 2021 by 2050, IEA, Paris, licensed under CC BY 4.0.

In the maritime sector, alternative fuels under consideration include liquefied natural gas (LNG), hydrogen, ammonia, methanol, and biofuels, where their compatibility and replacement of the current conventional marine fuel are summarized in Table 1. Among these alternatives, biofuels offer a particularly direct pathway to reducing carbon intensity (CI) and GHG emissions [4]. Its role in the global energy system is expected to expand significantly in the coming decades. According to the International Energy Agency’s Net Zero Emissions (NZE) Roadmap, the transport sector is projected to reduce its CO_2_ emissions to 0.7 Gt by 2050 (see Figure 1). Within this framework, the share of advanced liquid biofuels in transportation is expected to rise dramatically, from less than 1% in 2020 to approximately 45% by 2030 and 90% by 2050 [2]. In addition to biofuels, recycled carbon fuels (RCFs) represent another promising pathway for delivering lower-carbon energy alternatives. RCFs are derived from waste materials (plastics mainly), that contain fossil-based energy, which would otherwise remain unused or be inefficiently exploited [5]. RCFs are included under the latest EU climate policy, specifically within the Renewable Energy Directive (RED III, European Union, 2025), which classifies them among the advanced fuels from non-biomass feedstock [6]. Plastic waste is the main representative feedstock for the production of RCFs. While the pyrolysis of plastics has long been studied as a waste management strategy, its role in sustainable fuel production has gained increasing attention in recent years [5]. This is largely due to the rapid growth in global plastic production, which rose from 370.7 million tons in 2018 to 413.8 million tons in 2023, with fossil-based plastics accounting for 90.4% of the total [7]. Such figures highlight the urgency of shifting from a linear, fossil-fuel-based feedstock model toward a circular plastic economy.

**Table 1:** Comparison of current marine fuel oil (HFO) and replacement options; (+) refers to a beneficial value for the property, e.g., low flammability or high energy density, and (−) refers to a detrimental value [4].

	HFO ^a^	LNG ^b^	H_2_	NH_3_	Methanol	Biofuel
Flammability	+	+	−	−	+	+
Toxicity	+	+	+	−	−	+
Energy density	+	+	−	−	+	+
Carbon emissions	−	−	−(+)	−(+)	−(+)	+
Sulfur content	−	+	+	+	+	+
Nitrogen content	−	+	+	−	+	+
Compatibility with the current fleet and infrastructure	+	−	−	−	−	+

^a^ Heavy Fuel Oil, ^b^ Liquefied Natural Gas.

### 1.3. Thermochemical Production of Bio-Oils and RCF

**Figure 2:**
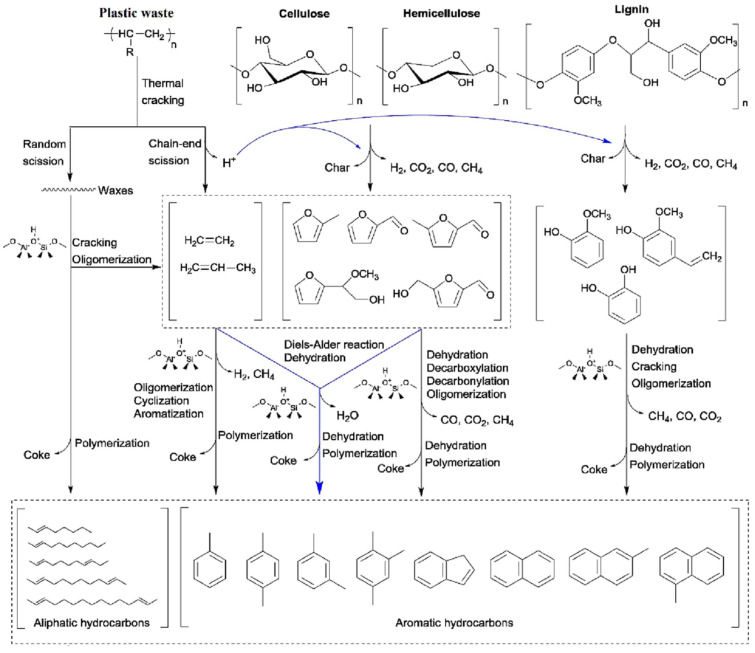
Proposed reaction pathways for the conversions of lignocellulosic biomass and plastics in co-feed catalytic pyrolysis using ZSM-5 (catalyst attack represented in blue lines); data obtained from [26].

One of the most promising alternatives to fossil fuels, including maritime transport, is biofuel derived from biomass [8]. These advanced biofuels are typically produced from agricultural and forestry residues, which are widely available and do not compete with food production. Biomass can be transformed into bioenergy through various technological routes, generally classified as thermochemical, biochemical, and chemical processes [9,10]. Thermochemical processes are especially useful for turning plant-based materials like cellulose, hemicellulose, and lignin, collectively referred to as lignocellulosic feedstocks, into liquid fuels [11]. Hemicellulose and cellulose are made up of sugar units, while lignin is composed of aromatic units (see Figure 2). A widely studied thermochemical route is pyrolysis, which proceeds in the absence of oxygen. Depending on the heating rate, reaction temperature, and residence time, pyrolysis can be categorized as slow, fast, or flash. Of these, fast pyrolysis is considered the most economically attractive. It operates at high heating rates (10–200 °C/s), moderate temperatures (400–600 °C), short residence times (<5 s), and rapid vapor cooling [12,13]. Under such conditions, it achieves the highest liquid oils yields while retaining most of the feedstock’s energy in the liquid fraction [13]. The commercial relevance of this pathway is underscored by initiatives such as Envergent (a joint venture between UOP/Honeywell and Ensyn), which has developed and deployed Rapid Thermal Processing (RTP^TM^) technology for biomass-to-fuel conversion via fast pyrolysis. Beyond the initial pilot-scale demonstration plant in Hawaii, Envergent’s technology has progressed toward commercial implementation, notably through its application at the Côte-Nord fast pyrolysis facility in Quebec, Canada, which has been operational since 2018 and converts woody biomass into pyrolysis oil. These developments highlight the increasing technological maturity and industrial deployment of fast pyrolysis pathways for renewable fuel production [14,15].

Due to differences in feedstocks, the liquid products derived from biomass are described in the literature under several distinct designations, including pyrolysis oil, pyrolytic liquid, pyroligneous acid, wood distillate, biocrude oil, and wood oil [16,17]. It is important to note that numerous reviews [8,18,19] have already addressed the production of biodiesel from lipid-rich feedstocks via transesterification. Oleaginous biomasses are mainly composed of lipids, particularly triglycerides, which fall outside the scope of this study. Instead, the present review focuses exclusively on the production of bio-oils from thermochemical processes. For consistency, the term bio-oil will be adopted throughout this work to refer collectively to the complex mixture of oxygenated organic compounds, such as acids, aldehydes, ketones, phenols, aromatics, alcohols, ethers, esters, carbohydrates, and heavy long-chain molecules, that typically constitute this product [16,20,21,22]. An in-depth analysis of bio-oil composition has become possible due to the development of advanced analytical techniques. In this context, Torri et al. [23] employed both GC/QMS and comprehensive two-dimensional GC coupled with time-of-flight mass spectrometry (GC × GC/TOF-MS) to characterize the complex mixture of lignocellulosic feedstocks (hardwood and softwood). The latter technique proved essential for resolving overlapping compounds, enabling the clear identification of characteristic markers of bio-oils. Furthermore, the study revealed that softwood-derived bio-oils, particularly those produced via fast pyrolysis, contained higher proportions of aromatic hydrocarbons, suggesting promising potential for fuel applications [23].

Interestingly, beyond lignocellulosic biomass, pyrolysis is also used for the conversion of plastic-derived feedstocks, into transport-grade fuels (RCFs), since their thermal decomposition can yield hydrocarbon-rich liquids with calorific values comparable to conventional fuels. Polymers can be broadly categorized into two major classes: thermoplastics and thermosetting. All thermoplastics like polyethylene (PE), polypropylene (PP), and polystyrene (PS) and heteroatom-containing polymers like polymethyl methacrylate, PMMA; polyesters, PES, and polyvinyl chloride PVC [24] offer advantageous characteristics compared to biomass as they yield pyrolysis oils rich in hydrogen and contain almost no oxygen. In polymers, cracking generally occurs through depolymerization, random chain scission, or the elimination of small molecules. The underlying mechanism, described in detail elsewhere [25], proceeds via radical chain reactions, involving three steps: initiation (formation of the first radicals), propagation (transformation of intermediate radicals), and termination (radical recombination). Notably, these classes of reactions help explain the pyrolysis behavior of different polymer types.

### 1.4. Challenges of the Direct Use of the Produced Oils in Shipping Engines

The large-scale adoption of biofuels in shipping still faces significant challenges. Recent studies show that while biofuels can substantially reduce emissions [14,27,28,29,30], ship engines are not easily adapted to new fuel types. Modifying engines and fuel infrastructure is costly, making it essential that marine biofuels conform to the specifications of conventional fuels. Such “drop-in” fuels can be used in existing systems without requiring major technical changes [14]. Medium- and slow-speed marine diesel engines are generally fuel-flexible and capable of operating on low-grade fuels. However, the direct use of bio-oils in diesel engines presents several challenges due to their unfavorable properties. The high heating value (HHV) of bio-oils is typically about half that of petroleum-derived heavy oils, primarily due to their elevated content of oxygenated compounds and water, which limit their direct application as transportation fuels. Solantausta et al. [31] investigated the direct injection of bio-oil in a high-speed, single-cylinder engine and reported that the bio-oil could not achieve auto-ignition without the use of additives. Moreover, its ignition delay was found to be 9 crank angle degrees (CAD) compared to 6 CAD for conventional No. 2 marine fuel oil, signifying that raw pyrolysis oils are not directly suitable for medium- or high-speed diesel engines and may be limited to use in low-speed applications unless upgraded.

**Figure 3:**
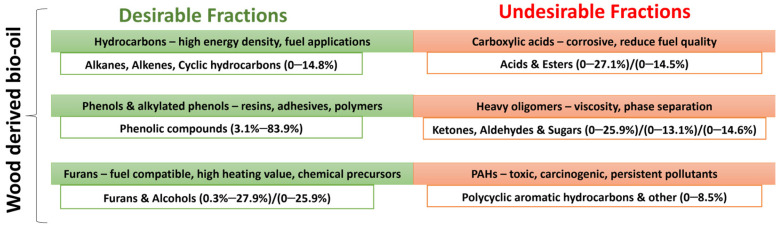
Schematic representation of the main fractions present in pyrolytic bio-oil and their respective peak area (in relative %) across different GC-MS analyses; reproduced from ref. [34,35].

In addition, due to high oxygen content, pyrolysis oils are characterized by high chemical instability, corrosiveness, and storage challenges. Among these oxygenates, lignin-derived heavy fractions contribute substantially to the high oxygen content. Acids are particularly problematic because of their high corrosivity, while carbonyls and heavy fractions promote chemical instability, phase separation, and the poor storage behavior of bio-oil (see Figure 3). The high reactivity of certain compounds promotes the formation of larger molecules, which in turn increases viscosity and slows down the combustion process [22]. Another challenge arises from the formation of polycyclic aromatic hydrocarbons (PAHs), which are persistent organic pollutants associated with toxicity, carcinogenicity, and environmental hazards [32]. Butler et al. [33] summarized the properties of oils obtained from laboratory-scale fast pyrolysis reactors using various feedstocks and emphasized the necessity of a catalytic upgrading step to enhance the fuel quality, concluding that their reduction or elimination is therefore critical for safe large-scale deployment.

From a marine fuel perspective, hydrocarbons represent the most desirable component, as they enhance the energy density and combustion properties of the product. Certain oxygenated compounds, such as phenol and alkylated phenolic derivatives, also hold significant commercial value. Strategies aimed at reducing acidic lignin-derived oxygenates while simultaneously enhancing hydrocarbon yields are essential to increase the HHV and overall fuel quality of bio-oil [32]. Over the past decade, significant research efforts have been devoted to improving the quality of bio-oil and developing upgrading technologies to address its inherent limitations, with the ultimate goal of enabling its direct utilization in internal combustion engines as a sustainable alternative to conventional fossil fuels. Such upgrading is essential to ensure compliance with existing standards, including ISO-8217 for marine fuels [14].

## 2. Upgrading of Bio-Oils and RCF

### 2.1. Catalytic Cracking

To steer the composition of bio-oil toward a higher proportion of desirable fractions, while suppressing the formation of undesirable compounds, a catalytic refining step can be introduced. This process involves the incorporation of an appropriate catalyst either directly into the pyrolysis feed or within an external catalytic bed, where the pyrolysis vapors are converted before condensation, following a catalytic cracking (CC) approach. The key distinction between these two configurations lies in their mode of contact and operating conditions: in the first (in situ) configuration, the catalyst directly interacts with the feed under the same pyrolysis environment, whereas in the second (ex situ) configuration, the catalyst operates in a separate reactor, allowing for more precise control over the reaction parameters and thus greater flexibility in optimizing the upgrading process [24].

**Figure 4:**
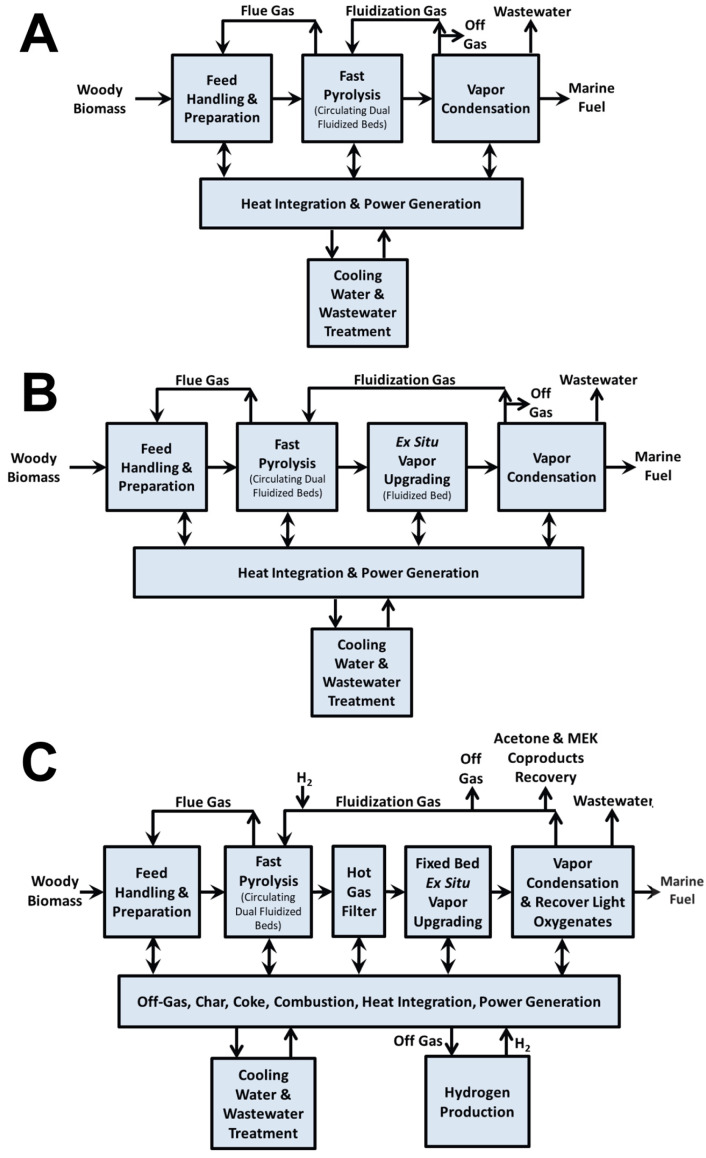
Process flow diagram and process description of fast pyrolysis: (**A**) fast pyrolysis (FP1), (**B**) catalytic fast pyrolysis with ZSM-5 catalyst in a fluidized bed (FP2), (**C**) catalytic fast pyrolysis with Pt/TiO_2_ catalyst in a fixed bed (FP3) for marine fuel production; data obtained from Supplementary Materials in Li et al. [42].

During catalytic cracking (CC), the deoxygenation of bio-oil primarily occurs through hydrogen transfer reactions, which enhance the hydrogen content of the resulting product. This process facilitates a variety of simultaneous and consecutive reactions, including hydrodeoxygenation, hydrocracking, polymerization, decarboxylation, decarbonylation, and hydrogenation [36]. Collectively, these reactions contribute to the conversion of oxygenated and heavy compounds into lighter hydrocarbons with improved stability and energy density. The role of the catalyst in CC is to promote the removal of most of the oxygen in selective ways and convert the active species to stable and useful components in bio-oil [13]. Among the heterogeneous catalysts investigated for this purpose, zeolites and aluminosilicates have emerged as the most extensively studied materials due to their well-defined pore structures, high surface acidity, and tunable physicochemical properties that promote both cracking and deoxygenation pathways [36,37,38,39,40,41]. In a recent techno-economic analysis (TEA), three processing routes for a feed mixture of forest residues and clean pine were evaluated: fast pyrolysis (FP), catalytic fast pyrolysis (CFP) over ZSM-5 zeolite, and CFP over Pt/TiO_2_ with co-fed hydrogen [42]. The corresponding process flow diagram is presented in Figure 4 of this review. The results highlight significant differences in product quality. Bio-oil obtained via FP exhibited a high oxygen content (49%), whereas both catalytic routes reduced the oxygen content considerably, achieving 17%. Other key properties, such as the lower heating value (LHV) and density, were also compared. For additional context, the study provided a summary of bio-oil characteristics obtained from alternative thermochemical conversion technologies beyond pyrolysis like the hydrothermal liquification (HTL) of sludge and manure (see Table 2). Overall, the findings indicate that compared with FP, CFP produces bio-oils with greater stability, attributed to the catalytic deoxygenation process. However, these catalytic bio-oils remain immiscible with conventional petroleum-derived fuels, limiting their direct application as drop-in fuels. Consequently, an additional hydrotreating step is required to further upgrade the oil and enable compliance with marine fuel standards.

CC can be applied to multiple biomasses and residues including plastics. Several studies have demonstrated the potential of this approach. For example, Serra et al. [43] addressed the environmental challenge of plastic waste and oil sludge, both containing mixtures of aliphatic and aromatic hydrocarbons, by employing pyrolysis (thermal and catalytic) to convert these residues into biofuels. Lin et al. [44] further highlighted that the decomposition of polyolefins into olefins and lighter paraffins typically requires higher temperatures (above 700 °C), which are significantly greater than those used in biomass pyrolysis. Such limitations point to the need for catalytic assistance. When catalysts are introduced, polymers can decompose at lower temperatures and with shorter reaction times while also achieving improved product selectivity and the inhibition of undesirable compounds.

Numerous studies have confirmed that thermo-catalytic co-pyrolysis of feedstocks with different chemical natures can enhance both the yield and quality of the resulting pyrolysis liquids. In particular, co-pyrolysis of plastics with biomass has been shown to enhance oil quality, as the hydrogen-rich polymers compensate for the oxygenated nature of lignocellulosic feedstocks [45]. For instance, blending lignocellulosic biomass with polyolefins increases the liquid fraction, improves calorific value, and reduces oxygenated compounds compared with biomass alone [46,47]. Catalysts also influence product distribution: acid catalysts, in particular, shift the spectrum toward long-chain hydrocarbons and aromatics. The benefits of catalytic co-pyrolysis have been experimentally validated. Rahman et al. [48] reported that the co-pyrolysis of pine and high-density polyethylene (HDPE) enhanced the formation of light hydrocarbon fractions and improved liquid quality, depending on the feed ratio, temperature, and catalyst type.

Similarly, the co-pyrolysis of oil sludge with polyolefins produced pyrolytic liquids rich in light hydrocarbons and with calorific values comparable to conventional fuel oil, demonstrating the feasibility of valorizing two problematic residues in a single thermo-catalytic process [49]. The synergistic improvements observed in catalytic co-pyrolysis are generally attributed to hydrogen transfer from plastic-derived radicals to oxygenated biomass fragments, as well as to catalytic cracking that promotes deoxygenation and fragmentation toward gasoline- and diesel-range hydrocarbons [47,48]. As illustrated in Figure 2, lignocellulosic components (cellulose, hemicellulose, lignin) primarily decompose into oxygenated intermediates such as furans, phenolics, acids, and ketones, while polyolefins undergo radical chain scission to generate long-chain olefins and hydrogen-rich fragments. The interaction of these streams under catalytic conditions explains the observed product synergies. Within this framework, the acid sites of zeolite catalysts (represented in blue lines) play a central role by directing stabilized intermediates toward gasoline- and diesel-range hydrocarbons through cracking, deoxygenation, and aromatization. At the molecular scale, furans can undergo decarbonylation, aromatization, and oligomerization inside the zeolite pore network, while phenolic compounds are converted into aromatics mainly through dehydration, cracking, and subsequent oligomerization [26].

**Table 2:** Marine bio-oil characteristics obtained from different thermochemical processes [42].

	SHTL_1_	SHTL_2_	SHTL_3_	MHTL_1_	MHTL_2_	MHTL_3_	FP_1_	FP_2_	FP_3_	LGFT	LEO
Fuel Production ^a^	33.24	31.39	31.72	32.38	32.86	33.46	52.82	31.07	34.48	41.28	56.38
Fuel yield ^b^	107.76	101.74	102.83	101.36	106.52	108.46	72.94	42.90	47.61		77.86
Energy Efficiency	71%	67%	63%	66%	61%	58%	64.7	38.0%	42.2%	52%	47%
Carbon Efficiency	72%	67%	63%	67%	65%	63%	61.9	33.2%	35.7%	63%	61%
Fuel Properties:											
Density (g/mL)	0.98	0.96	0.96	0.96	0.96	0.96	1.20	0.97	0.97	0.83	1.25
Carbon Content	75%	85%	86%	72%	84%	85%	44%	76%	75%	82%	54%
S (wt%)	1.11	0.39	0.00	0.70	0.24	0.01					
O (wt%)	4.8%	2.5%	1.0%	14.0%	5.0%	0.5%	49.0%	17.0%	17.0%		41%
LHV (Btu/gal)	124,630	148,407	149,611	113,947	146,417	146,665	71,570	110,454	112,735	128,154	96,804
LHV (MJ/kg) ^c^	35.445	43.087	43.437	33.082	42.509	42.581	16.623	31.738	32.393	43.035	21.585

^a^ Million Heavy Fuel Oil Gallon Equivalent (MMHFOGE)/year, ^b^ Heavy Fuel Oil gallon equivalent (HFOGE)/dry ton biomass, ^c^ Calculated based on the respective densities, Sludge HTL (SHTL_1_-STHL_2_-STHL_3_), Manure HTL (MHTL_1_-MHTL_2_-MHTL_3_), Fast Pyrolysis (FP_1_), Catalytic Fast Pyrolysis with ZSM-5 catalyst in a fluidized bed (FP_2_), Catalytic Fast Pyrolysis with Pt/TiO_2_ catalyst in a fixed bed (FP_3_), Landfill Gas Fischer-Tropsch (LFGFT), Lignin Ethanol Oil (LEO).

### 2.2. Hydrodeoxygenation

Another crucial upgrading route is catalytic hydroprocessing (hydrodeoxygenation, HDO), wherein the condensed bio-oil liquids are treated with a hydrogen donor, typically molecular hydrogen (H_2_) or a hydrogen-rich solvent, in the presence of suitable catalysts. During this process, water is generated as a by-product of the reaction between hydrogen and oxygen-containing compounds, and at higher levels of deoxygenation, distinct organic and aqueous phases commonly appear due to changes in the polarity and composition of the reaction products [50]. Achieving complete deoxygenation requires severe operating conditions, generally involving elevated hydrogen pressures (75–300 bar) and high hydrogen consumption rates. Depending on the complexity of the functional groups present in bio-oils, Grange et al. [51] reported that hydrogen consumption during hydrodeoxygenation varies significantly, from approximately 2 molecules of H_2_ per group for ketones to 8 molecules of H_2_ per molecule for dibenzofuran, highlighting the differences in apparent reactivity. Under such conditions, oxygenated species undergo hydrodeoxygenation reactions, leading to the removal of oxygen atoms and the stabilization of the bio-oil. The catalysts developed in this context usually contain transition or noble metals combined with acid-activated carriers, where they are reviewed elsewhere [8,50,52]. However, in the preparation, the high cost of conventional precious metal catalysts has led to increased attention on alternative catalysts with comparable performance.

In a recent study, Chen et al. [4] successfully combined catalytic fast pyrolysis (CFP) of woody biomass using ZSM-5 (Si/Al = 30) with subsequent mild hydroprocessing at the bench scale to enhance fuel properties. The upgraded heavy fraction (~90% of the CFP oil) exhibited a dramatic reduction in oxygen content, from approximately 23% to below 5% under mild conditions, and to ~0.05% under severe treatment, while maintaining good miscibility with very low sulfur fuel oil (VLSFO). Following hydrotreating and distillation, the oil fractions satisfied most ISO-8217 specifications, including viscosity, flash point (>60 °C), and negligible sulfur content (<0.5%), with densities ranging from 950 to 1020 kg·m^−3^ (slightly exceeding the specification under less severe conditions). Moreover, a techno-economic analysis revealed that moderate hydrotreating could significantly reduce hydrogen demand and catalyst requirements, thereby lowering the total processing costs by up to 30% compared to more severe upgrading conditions.

### 2.3. Limitations of Zeolite Catalysts and the Potential of Montmorillonite Clays

Despite the significant improvements achieved through zeolitic catalytic pyrolysis, the long-term stability of zeolite catalysts remains a major challenge. Coke and tar deposition, together with structural degradation under thermal conditions, can severely limit catalyst lifetime and efficiency. In zeolites, coke and tar deposits block pores and cover acid sites, reducing both activity and selectivity, while dealumination under steam conditions can lead to irreversible loss of acidity [53]. Partial hydrolysis of the framework in the presence of steam extracts aluminum atoms, generating structural defects with hydroxyl groups, which reduces crystallinity, alters acidity, and diminishes catalytic performance [52].

For instance, catalytic pyrolysis of pine performed in a fluidized bed reactor at 450 °C using different zeolite topologies (H-Beta-25, HY-12, HZSM-5-23, and HMOR-20) demonstrated pronounced variations in coke deposition related to acidic site strength. HY-12 exhibited the highest coke content (16.7 wt%), attributed to its large pore cavities (11.8 Å) and high surface area (884 m^2^·g^−1^) [54]. Structural features such as pore size, topology, and crystallite dimensions influence both the extent and type of deactivation. Larger pores facilitate the diffusion of bulky oxygenates but also promote secondary reactions that accelerate coke formation. Two principal types of coke are generally distinguished: thermal coke, formed on the external surface via polymerization of phenolic compounds, and catalytic coke, deposited within micropores through aromatization, oligomerization, condensation, and cyclization of oxygenates [55]. Carlson et al. [53] further demonstrated that catalytic deactivation of HZSM-5 during pine sawdust pyrolysis primarily affected non-framework Lewis acid sites, whereas strongly bound ammonia on Brønsted acidity remained largely intact.

Such limitations, combined with the relatively high cost and limited feedstock flexibility of zeolites, including industrial grades of USY and ZSM-5, which are often priced in the tens to hundreds of dollars per kilogram range, underscore the need for alternative catalysts capable of operating under milder upgrading conditions while maintaining performance.

In this context, naturally abundant montmorillonite clays have emerged as promising alternatives. MMT-based catalysts offer tunable acidity, larger interlayer spacing, and enhanced resistance to coking and deactivation, particularly under mild catalytic cracking and HDO conditions. These clay materials are significantly cheaper at the raw material level, with laboratory prices on the order of tens of dollars per kilogram and the potential for even lower costs at industrial scale. This inherent cost advantage, coupled with potential improvements in long-term stability and feedstock flexibility, supports the investigation of clay-based catalysts as cost-competitive alternatives for bio-oil and RCF upgrading.

From a comparative standpoint, zeolites and MMT-based catalysts exhibit fundamentally different catalytic behaviors. Zeolites are characterized by strong Brønsted acidity and well-defined microporous frameworks, which favor deep deoxygenation and aromatization but also accelerate secondary reactions leading to rapid coking and deactivation, particularly when processing oxygen-rich or water-containing feeds. In contrast, montmorillonite clays possess weaker and more tunable acidity, predominantly Lewis-type sites, and a layered mesoporous structure that facilitates the diffusion of bulky intermediates. These features generally result in lower coke formation rates and improved tolerance toward complex, heterogeneous feedstocks, albeit at the expense of lower intrinsic deoxygenation severity under comparable conditions. Consequently, while zeolites remain highly effective for aggressive upgrading, MMT-based catalysts emerge as complementary alternatives better suited for mild upgrading strategies prioritizing stability, feedstock flexibility, and cost efficiency.

To the best of our knowledge, this review is the first to address the production of marine fuel feedstocks through an integrated processing framework based on MMT-derived catalysts. The following section explores these aspects in detail.

## 3. Montmorillonite-Based Catalysts for Bio-Oil and RCF Upgrading

### 3.1. Structure, Compositions, and Properties

**Figure 5:**
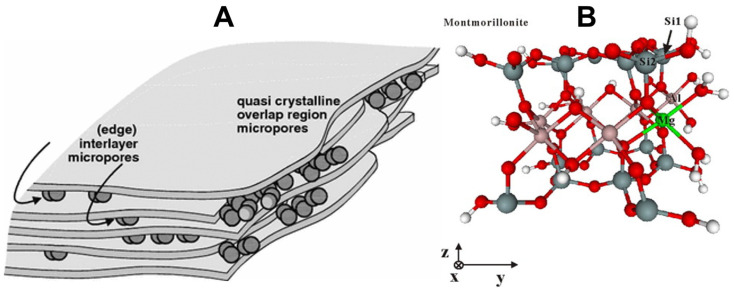
Schematic representation of microporosity and edge surface model of MMT, (**A**) microporosity types, (**B**) edge surface model with the solution regions (20 A°) in the direction vertical to the edge surfaces (y direction). O = red, H = white, Si = grey, Mg = green, and Al = pink. Si2 connects with Mg via a bridge oxygen atom while Si1 connects with Al; data adapted from [66,67].

The catalyst type has emerged as a central research focus at this stage of this study. In this context, alternative catalysts, such as naturally abundant clays, have emerged as low-cost, environmentally benign promising alternatives to conventional zeolitic systems. Clays are layered minerals with the structure and the texture determined by the stacking of phyllosilicate sheets, held together either by electrostatic interactions, as in smectites, vermiculites, and micas, or by hydrogen bonding, as in kaolinites [56]. Clay rocks usually contain non-clay constituents such as feldspar, quartz, and carbonates, which are typically concentrated in the coarser (>2 μm) fraction, organic matter, and nm-sized inorganic fractions [57]. Smectite clays have medium to low layer charge density (1.2–1.8 a.u), and the most common representative of the smectite group is montmorillonite (MMT), where it serves as a model material for studying clay-based catalysis. It belongs to the family of 2:1 (or TOT) dioctahedral layered silicates, in which an octahedral sheet is sandwiched between two tetrahedral sheets oriented inward. Linkage between adjoining tetrahedra occurs through corner sharing, while those in the octahedra sheet occur through edge sharing [58]. The idealized composition of MMT can be expressed as: $M_{x}^{+}.nH_{2}O\left({Al}_{2-x}{Mg}_{x}\right)\left({Si}_{4}\right)O_{4}{\left(OH\right)}_{2}$, where $M_{x}^{+}$ represents exchangeable interlayer cations (${Na}^{+}$, ${Ca}^{2+})$, and the layer charge arises from the partial substitution of counterion ${Mg}^{2+}$ for ${Al}^{3+}\;$in the octahedral sheets giving rise to the dioctahedral character [59]. MMT display a turbostratic arrangement of the phyllosilicate sheets, responsible for imparting their microporosity, located at the quasi-crystalline edges of the material and arising from imperfections in the stacking of individual TOT layers, leading to the formation of overlap regions between adjacent layers (see Figure 5) [60]. Various mineralogical classifications of smectites and clay-based materials have been extensively documented in the literature [58,61,62,63]. However, for the sake of conciseness, the present discussion will focus primarily on the surface acid–base properties of montmorillonite (MMT), as these characteristics play a decisive role in governing the catalytic reactions occurring on its surface.

The acidity of MMT arises from two principal sources: (i) interlayer charge-balancing cations, which exert a strong polarizing effect on coordinated water molecules but are generally less accessible, and (ii) specific sites located at the layer edges [64]. From a surface chemistry perspective, Filimonova et al. [65] demonstrated that Brønsted acid sites are associated with surface hydroxyl groups, particularly aluminol (Al-OH) and silanol (Si-OH) moieties situated at defect sites, whereas undercoordinated Al^3+^ ions function as Lewis acid sites. They further highlighted that the removal of surface-adsorbed water and dehydroxylation enhance Lewis acidity, thereby altering the overall acid–base properties of the mineral. Notably, the basal surfaces of clays are considered largely pH-independent, while edge surfaces exhibit more complex structures and pH-dependent reactivity. Complementing this view, Lui et al. [66] proposed a structural model of the MMT (010) type plane interface (see Figure 5), in which Brønsted sites include Si-OH groups, coordinated water ligands on Al-OH-OH_2_ moieties, and Mg-(OH_2_)_2_ groups, whereas Lewis sites correspond to oxygen atoms of Si-OH and hydroxyl groups of Al-OH-OH_2_. Together, these findings reveal that MMT exhibits dual acidic functionality (Brønsted and Lewis), governed by both interlayer cations and edge structural features. Although edge surfaces account for only about 10% of the total surface area of MMT, their contribution is sufficient to exert a significant influence on the overall acidity of the material [58,64].

### 3.2. Modification of MMT

Many catalytic reactions over MMT occur at external particle surfaces or within intradomain regions, including interparticle pores and interlayer spaces. Consequently, pretreatments that enhance the accessible surface area and porosity of the clay are particularly beneficial for improving catalytic activity. Such textural modifications can be introduced through acid activation, thermal treatment, ion exchange with metal cations, or pillaring. In what follows, these modification strategies will first be examined in detail to better understand how they influence the physicochemical and catalytic properties of MMT. Building on this foundation, the subsequent section will review the performance of both pristine and modified montmorillonites in biofuel production.

#### 3.2.1. Acid-Activated MMT

Among the different modification strategies, acid activation remains the most widely employed method for enhancing the catalytic performance of MMT. The process involves ion exchange, whereby interlayer metal cations are replaced by protons (H^+^), leading to the formation of abundant Brønsted acid sites within the interlayers [8]. At the same time, partial leaching of Al^3+^ ions from the octahedral sheet further intensifies the acidity of the material. The introduced protons first attack the edge surfaces of clay particles before diffusing inward, eventually dissolving portions of the octahedral sheet [68]. This treatment not only increases the number and strength of acid sites (see Table 3) but also removes structural impurities, thereby improving pore volume, specific surface area, and overall catalytic activity. Nevertheless, excessive acid treatment can damage the structural integrity of MMT, ultimately diminishing its catalytic efficiency. Acid activation has been carried out to date using a variety of mineral acids, such as HCl, H_2_SO_4_, HNO_3_, and H_3_PO_4_ [68,69,70]. Organic acid modifications have also been reported, though these are outside the scope of this review. On the Hammett scale (H_0_), replacing the native counterions with protons provides a particularly effective means of enhancing acidity. For example, the conversion of Na-MMT (Na^+^ as interstitial cation) to H^+^/Al^3+^-MMT (exchanged forms) or into K10 (commercial) through acid treatment significantly increases the surface acidity, with H_0_ values shifting from approximately +2 to as low as −8, depending on the severity of treatment [62].

**Table 3:** Surface properties of commercial K-catalysts [58].

Catalyst	SSA ^d^ (m^2^/g)	APD ^e^ (nm)	PV ^f^ (mL/g)	CEC ^g^ (cmol/kg)	Acidity Brønsted ^h^ (mmol/g)	Acidity Lewis ^h^ (mmol/g)
MMT ^a^	88	4.4	0.097	91		
Commercial KSF ^b^	9–40	5.0	0.011		0.59	0.15
Commercial KSF/0 ^c^	117	7.4	0.215		1.03	0.20
Commercial KP10 ^c^	169	7.1	0.300		0.49	0.09
Commercial K10 ^c^	229–254	5.6	0.320		0.33	0.29
Commercial K0 ^c^	268		0.380			
Commercial KS ^c^	322		0.465		0.45	0.26
Cu^2+^-K10	236			39	0.13	0.68
Zn^2+^-K10	213			36	0.16	0.73
Al^3+^-K10	234		0.293	30		
Fe^3+^-K10	239			54	0.35	0.34

^a^ The parent clay is a montmorillonite-rich bentonite. ^b^ KSF was obtained by treating the parent material with H_2_SO_4_ at room temperature. ^c^ K-catalysts were formed by treatment with HCl of variable concentrations at 80–90 °C. ^d–g^ Accounts for specific surface area, average pore diameter, pore volume, cation exchange capacity, respectively. ^h^ Determined by adsorption of NH_3_ and pyridine combined with infrared spectroscopy (FTIR).

**Figure 6:**
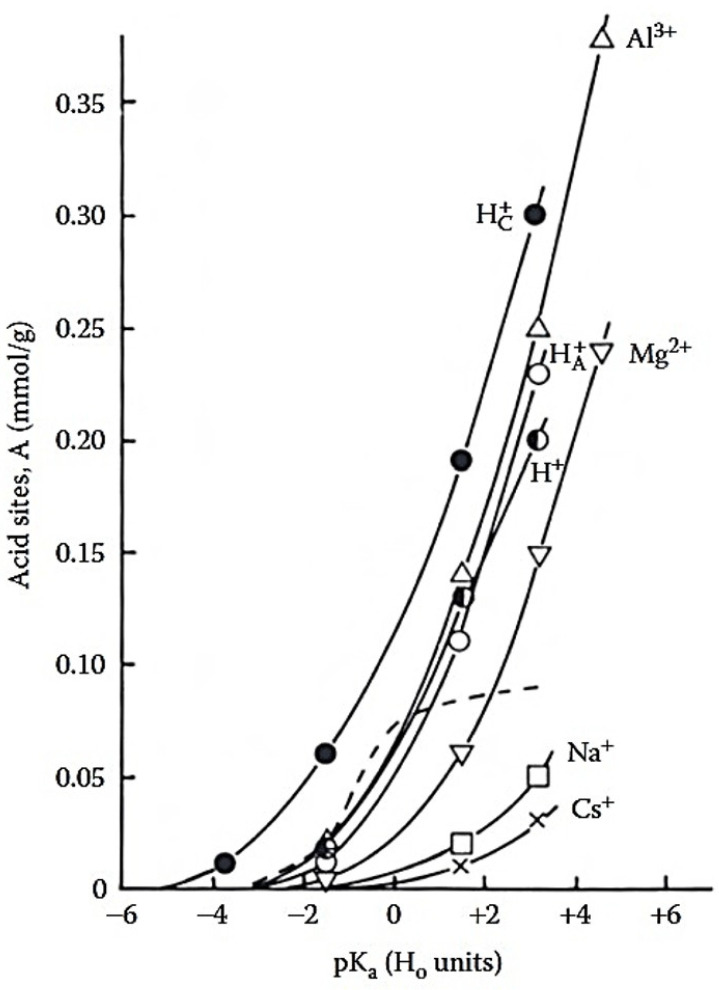
Acid strength distribution curves for MMT saturated with different counterions, determined by titration with *n*-butylamine. The dashed line represents the parent MMT; data adapted from [58].

Importantly, acid activation underpins the development of several industrially relevant MMT-based catalysts, including the K-catalysts (Süd-Chemie) and the Filtrol series, whose main characteristics are summarized in Table 3, and their activity in biofuel production is discussed below. These acid-activated (K-type) catalysts are valued for their high reactivity, enhanced surface acidity, and improved structural stability, making them effective and cost-efficient alternatives to conventional solid acids [62,63,68,71,72,73,74,75,76,77,78]. The extent of acidity in MMT is strongly influenced by the type of exchangeable cation. Figure 6 shows that both the total titratable acidity and the concentration of relatively strong acid sites were closely related to the ionic potential of the counterions [58]. The concentration of acid sites increased at H_0_ = +1.8, in the order: ${Cs}^{+}$-MMT < ${Na}^{+}$-MMT < ${Mg}^{2+}$-MMT < $H_{A}^{+}$-MMT < $H^{+}$-MMT < ${Al}^{3+}$-MMT < $H_{c}^{+}$-MMT, where $H^{+}$ denotes proton-exchanged MMT, $H_{A}^{+}$ refers to acid activated MMT, dried at 130 °C, $H_{c}^{+}\;$denotes acid activated MMT, dried under ambient conditions.

#### 3.2.2. Thermal Activation of MMT

Upon heating, MMT undergoes three distinct structural transformations. First, between room temperature and 300 °C, dehydration occurs through the loss of water from external particle surfaces, intraparticle pores, and the hydration shell of exchangeable cations. Second, above 400 °C, dehydroxylation takes place via condensation of structural hydroxyl groups, leading to the release of interlayer water. Finally, in the range of 600–900 °C, structural decomposition and recrystallization are observed [79].

These processes can significantly alter the texture of the clay, as dehydration often induces changes in layer stacking and porosity. For example, heating monovalent cation-exchanged MMT at 150 °C caused interlayer water loss and a reduction in basal spacing from 2 to 0.98 nm, signaling partial collapse of the interlayer region. In contrast, MMT exchanged with polyvalent cations resisted complete collapse even after heating at 300 °C [58]. The evolution of acidity with temperature is equally important for catalytic applications. A commercial acid-activated MMT (Fulcat 40) used for the rearrangement of α-pinene to camphene showed that Brønsted acidity reached its maximum at 150 °C, correlating with the highest reaction rate under these conditions. In contrast, when the same catalyst was employed for the conversion of camphene to isobornyl chloride, maximum Lewis acidity was achieved after heating at 300 °C, which coincided with enhanced catalytic performance [80]. This highlights the possibility of tuning the Brønsted/Lewis acid balance by controlled thermal treatment. K-type catalysts have similarly been applied in Friedel–Crafts alkylation, where both Brønsted and Lewis sites play synergistic roles [81]. In cation-exchanged K10, acidity is initially dominated by Brønsted sites but shifts progressively toward almost exclusively Lewis acidity after calcination at 500 °C [82]. For instance, in the Friedel–Crafts alkylation of anisole with dienes, calcination of K10 at 550 °C generated the optimal acidity profile for efficient catalysis [83].

#### 3.2.3. Pillared Interlayered MMT

**Figure 7:**
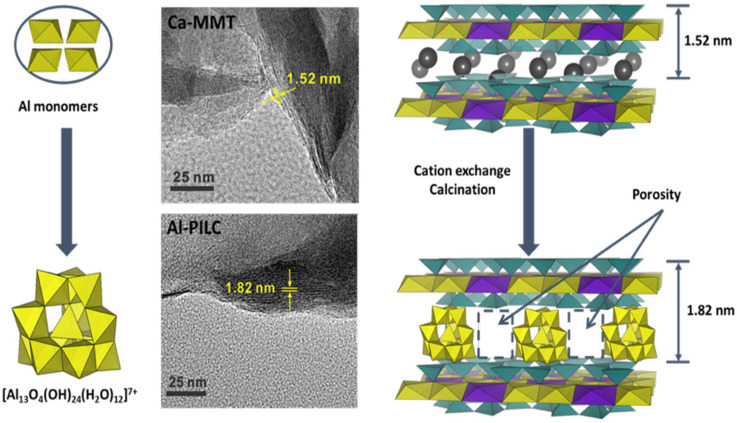
Schematic illustration of the main steps involved in the synthesis of Al-PILC from Ca-MMT, along with the corresponding TEM images. Reproduced from ref. [85], Copyright © 2017, with permission from Elsevier.

Pillared clays (PILCs) represent another important class of modified MMT, produced through topotactic reactions in which the parent clay framework is preserved but the interlamellar counterions are replaced with bulky polycations introduced from solution. Upon calcination, these polycations are converted into their corresponding metal oxides, which remain lodged between the silicate sheets and promote pillar-layer cross-linking [64]. Successful synthesis results in silicate platelets being permanently propped open, with covalent bonds formed to the tetrahedral sheets, thereby stabilizing an expanded structure [84]. Among the different pillaring agents, the best known is the aluminum-containing Keggin ion, [Al_13_O_4_(OH)_24_(H_2_O)_12_]^7+^, commonly abbreviated as (Al_13_)^7+^. Al-PILC has rigid, non-swelling, interlayer micropore structures (pore volume typically 0.1 cm^3^·mL^−1^), moderate surface areas, and the ability to retain microporosity at high temperatures [62]. For instance, Zhu et al. [85] synthesized Al-PILC by hydrolyzing an intercalating solution of 1 M AlCl_3_ with 0.6 M NaOH at an (OH^−^)/(Al^3+^) molar ratio of 2.4. The resulting (Al_13_)^7+^ polycations were then exchanged into a suspension of Ca-MMT (swelled in water), with the clay/Al_13_ ratio carefully controlled to improve complex stability. The obtained material exhibited a characteristic basal spacing of 1.82 nm, corresponding to an interlayer separation of 0.86 nm, considering the intrinsic thickness of an individual MMT layer (0.96 nm) (see Figure 7). After calcination at 300 °C for 2 h, the Al cluster was obtained without significantly reducing the basal spacing.

Both Brønsted and Lewis acidity have been evidenced in MMT. A central question that has subsequently emerged is whether this acidity arises primarily from the clay layers, from the inserted pillars, or from a combination of both. Comparative studies on different pillared clays, including montmorillonites, saponites, and beidellites (referred to as Al-PILM, Al-PILS, and Al-PILB, respectively) have shown that even with the same pillaring agent, the resulting materials may display markedly different acidic properties [59,64,86]. For instance, in the case of Al-PILB, Poncelet et al. [86] reported that its higher proportion of tetrahedral Al^(IV)^ in the clay layers led to an increased concentration of Brønsted sites compared with Al-PILM. Similarly, in Al-PILS, a distinct type of acid site was observed, associated with Si-OH-Al groups formed upon proton attack on tetrahedral Si-O-Al linkages [64]. This feature, absent in Al-PILM, explains the comparatively lower acidity of the latter. In dioctahedral clays such as MMT, vacant O_4_(OH)_2_ octahedral sites are available in the layer structure. However, the scarcity of tetrahedral Al atoms in Al-PILM prevents the formation of Si-OH-Al (pillar layer anchoring reaction), thus limiting the number of Brønsted sites [59]. Upon intercalation, Al_13_ Brønsted polyacids can transfer protons into these vacant octahedral sites, where they are more effectively stabilized. As a result, in Al-PILM, the inserted pillars contribute predominantly to Lewis acidity [87]. Beyond Al-PILCs, a broad range of polyoxo cations have been explored as pillaring agents, including Cr, Fe, Ga, Si, Ti, and Zr, as well as mixed combinations such as Al-Cr, Al-Fe, Al-Ga, Al-Si, and Al-Zr. These have been extensively reviewed in the literature [41,63,68,88,89,90,91,92]. 

A representative study by Ming et al. [93] showed that Na-MMT pillared with different metal oxides exhibits significant variations in acidity (see Table 4), with Ti-PILC displaying the highest acidity and Ni-PILC the lowest. Lower acidities were reported when Fe pillars were used, as well as in mixed systems such as Al/Zr and Al/Fe. Taken together, these observations indicate that the main source of Lewis acidity resides in the inserted pillars, since it directly depends on the nature and concentration of the pillaring species [84]. In contrast, Brønsted acidity is largely associated with the clay layers themselves, with only a minor contribution from the pillars. Consequently, the catalytic advantages of transition-metal-pillared clays are less related to an increase in Brønsted acidity and more attributable to their enhanced Lewis acid character.

**Table 4:** Acidity of different metal oxide pillared montmorillonite [84].

Catalyst	SSA (m^2^/g)	d_(001)_ (nm)	Acidity (μv)
Na-MMT	51	1.28	86
Al-PILC	190	1.73–1.89	425–442
Zr-PILC	191	1.82	570
Ti-PILC		1.5	620
Fe-PILC	109	1.55	340
Ni-PILC	58	1.48	228
Al/Zr-PILC		1.56	390
Al/Fe-PILC		1.58	340

Another effective strategy to enhance the Brønsted acidity of PILCs is to use acid-activated smectites as host materials, leading to the formation of pillared acid-activated clays (PAACs). Acid-activated clays alone were already discussed earlier, but their combination with pillaring agents provides an additional boost in acidity, typically accompanied by increases in both pore volume and average pore diameter. For instance, Mishra and Parida [94] compared chromia-pillared materials prepared from Na-MMT and from acid-activated MMT (H-MMT). At all calcination temperatures, Cr/H-PILC displayed higher total and Brønsted acidity than Cr/Na-PILC, clearly showing that acid treatment enhances the acidity of pillared clays. Similarly, Jones et al. [95] investigated the microstructure of Al-PILCs and their acid-treated counterparts. They observed that pillared acid-activated clays exhibit a higher pore volume, distributed over a broader pore size range, whereas conventional PILCs show more limited pore volumes and narrower pore size distributions. This difference was attributed to the lower cation exchange capacity (CEC) of acid-activated clays (58 meq/100 g) compared with parent clays (88 meq/100 g). With fewer interlayer cations available for exchange, less alumina is incorporated during pillaring, leaving more space in the interlayer region and resulting in larger pore diameters and higher pore volumes. These observations are consistent with the partial destruction of the layered structure of montmorillonite during acid treatment and the formation of amorphous silica, the extent of which depends on the severity of the acid attack [96].

### 3.3. Catalytic Cracking over MMT-Based Catalysts

#### 3.3.1. Applications in Biomass Pyrolysis

Unmodified MMT and bentonite clays have long been employed as model catalysts in biomass pyrolysis due to their moderate acidity and inherent structural stability. These materials, characterized by exchangeable interlayer cations (Na, Ca, Mg), provide weak Lewis acid sites that facilitate depolymerization while controlling condensation reactions. This allows for the stabilization of reactive intermediates and limits secondary char formation. In practical applications, bentonite-assisted pyrolysis of lignocellulosic residues, such as almond shells [97], cherry pits [98], peat [99], and avocado pits [100], has yielded liquid fractions ranging from 37% to 62%. Across these studies, a common observation is that pristine clays exhibit mild catalytic deoxygenation via dehydration and decarboxylation pathways, reflecting their limited acidity. The resulting bio-oils are typically of reduced viscosity and density, although the oxygen content remains relatively high (32%). Distillation profiles of these oils resemble those of diesel and gasoline, with enrichment in phenolics (34%), ketones (14%), and oxygenated aromatics (2%), as reported in Kar et al.’s study [97]. Investigations on pine woodchips [101] and macroalgae [102] further confirm that pristine MMT enhances condensable volatile retention and moderates thermal cracking, yielding stable bio-oils. Nonetheless, the limited number of Brønsted acid sites constrains deoxygenation, resulting in products enriched in phenolics and ketones, indicative of incomplete lignin-derived oxygen removal.

**Figure 8:**
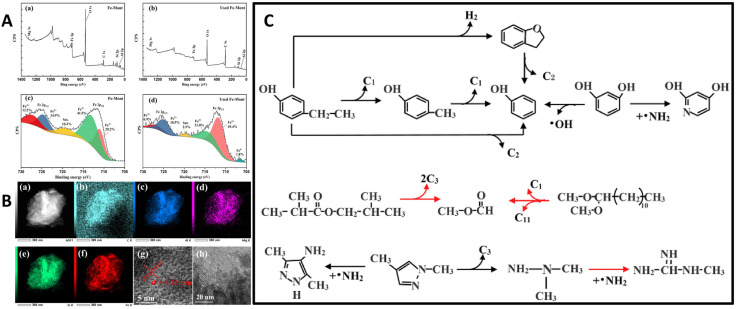
(**A**) The atomic ratio and chemical valence of elements present on the Fe-K10 surface using X-ray photoelectron spectroscopy (XPS), and deconvolution of the Fe_2p_ region of: (**a**–**c**) fresh and (**b**–**d**) used. (**B**) TEM and TEM-mapping images of Fe-K10: (**a**) Fe-K10 at the scale of 300 nm, (**b**–**f**) TEM-Mapping of C, Al, Mg, Si, Fe elements, respectively, (**g**,**h**) Fe-K10 at the scale of 5 and 20 nm. (**C**) Proposed reaction pathway of pyrolysis of Corncob on Fe-K10, where the chemicals are divided into three groups: phenolics, aliphatics, ring or chain structures with more than one nitrogen atoms (red arrow indicates the reactions promoted by Fe-K10). Reproduced from ref. [105], Copyright © 2024, with permission from Elsevier.

**Figure 9:**
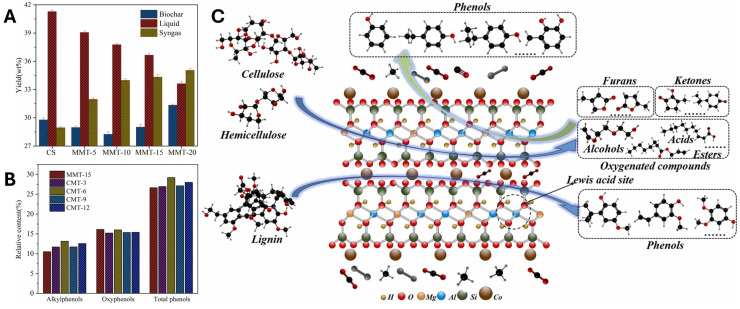
(**A**) Pyrolytic product distribution of corn stalk (CS) catalyzed by K10 at different mass fractions in the mixed sample (K10 and CS). (**B**) Relative content of phenols in the obtained bio-oils from pyrolysis of CS catalyzed by K10 at different mass fractions in the mixed sample (MMT and CS). (**C**) Proposed reaction pathway for the formation of phenols produced by the catalytic pyrolysis of corn stalk on Co-K10. Reproduced from ref. [107], Copyright © 2023, with permission from Elsevier.

Acid activation significantly improves the reactivity of MMT by introducing stronger acid sites and mesoporosity. This modification promotes more efficient cracking, dehydration, and decarboxylation, leading to deeper deoxygenation and higher-quality oils. For example, K10 clay has been reported to reduce the oxygen content of bio-oils and modestly decrease phenolic content relative to non-catalytic systems [103]. Further enhancements are achieved by incorporating transition metals (Fe, Co, Al) into acid-activated MMT. Ellison et al. [104] demonstrated that Fe-K10, applied to pine sawdust pyrolysis, minimized carboxylic acids and aldehydes while promoting ketonization and stabilizing pyrolytic liquids, where 90% of products were stable functional groups such as furans, ketones, and alkyl phenols. Fe species facilitated the hydrolysis of methoxy-phenols into alkyl phenols, although a reduction in liquid yield (31%) was observed compared to non-catalytic pyrolysis. More recently, Cai et al. [105] optimized Fe dispersion on K10, enhancing hydrogen evolution and secondary cracking of C_11_–C_20_ intermediates. This approach yielded lighter hydrocarbons and stabilized phenolic derivatives, achieving the highest liquid yields in their study (57%). The efficacy of Fe species is partly attributed to their reduction during pyrolysis, resulting in Fe^3+^ oxides that act as Lewis acids by accepting electrons from adsorbed organic compounds (see Figure 8) [58]. Similar improvements have been observed with Co-K10 and Al-K10, which enhance C-O bond scission and promote depolymerization while favoring phenolic formation via decarbonylation and decarboxylation [106,107]. On the other hand, cellulose and hemicellulose initially undergo thermal cracking, yielding primary oxygenated intermediates such as furans, ketones, and related compounds (see Figure 9). These intermediates subsequently transform into aromatic precursors containing benzene rings through sequential dehydration, dehydrogenation, and Diels–Alder cyclization reactions. The resulting intermediates then participate in further rearrangement and free-radical pathways, ultimately leading to the formation of phenolic compounds on Co-K10 [107]. Compared to pristine clays, acid-activated variants shift product selectivity from phenolic-rich fractions toward lighter oxygenates and hydrocarbons, increasing heating values. However, the absence of shape-selective micropores limits deep aromatic hydrocarbon formation relative to zeolites, maintaining a favorable balance between activity, selectivity, and low coke deposition.

**Figure 10:**
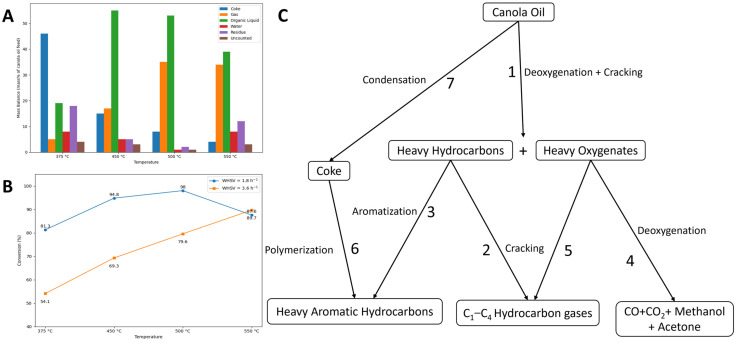
(**A**) Mass balances (mass% of canola oil feed) for the conversion of canola oil over Al-PILC at different temperatures at 1.8 WHSV, h^−1^. (**B**) Conversion of canola oil over Al-PILC at 1.8 and 3.6 WHSV, h^−1^. (**C**) Proposed reaction pathway for the conversion of canola oil over silica-alumina and Al-PILC catalysts. Reproduced from ref. [108], Copyright © 1995, with permission from John Wiley and Sons.

PILCs, such as Al-PILC, further refine this catalytic behavior. Al-PILC exhibits high selectivity toward light oxygenates and aliphatic hydrocarbons, favoring cracking reactions over cyclization and hydrogen transfer. Adjay et al. [108] highlighted that reaction pathways are influenced by pore size and interplanar spacing, with initial cracking occurring in the interlayers, followed by diffusion into pores for further cracking, deoxygenation, and aromatization. The open, uni-dimensional pore structure confines polymerization and coke formation primarily to the interlayer regions, with coke formation associated with abundant Lewis acid sites (see Figure 10).

While most previous studies have focused on the direct catalytic conversion of biomass, it is equally important to evaluate catalyst performance during the upgrading of pyrolysis bio-oils, which better reflects realistic processing conditions. In this context, the work of Fatimah et al. [109] provides a representative example, demonstrating that Al-PILC promotes pyrolytic oil degradation through cationic mechanisms facilitated by higher Lewis acid density, shifting product selectivity from C_7_–C_8_ hydrocarbons (guaiacol, methyl guaiacol) to smaller oxygenates (C_2_, C_6_, C_8_ compounds, e.g., acetic acid, phenol). Compared to pristine MMT, PILCs provide an optimal compromise between activity, stability, and selectivity, surpassing acid-only systems in both oil quality and yield consistency. The transition from pristine to pillared MMT reflects a progressive tuning of acidity and porosity, enabling deeper deoxygenation and higher hydrocarbon selectivity without sacrificing thermal stability. Nevertheless, the absence of hydrogenating functionality limits complete oxygen removal, necessitating downstream HDO to produce fuel-range hydrocarbons.

#### 3.3.2. Applications in Plastic Pyrolysis

In polymeric and mixed plastic systems, pristine MMT and bentonite clays exhibit moderate acidity, well-suited for controlled chain scission and hydrogen-transfer stabilization during pyrolysis. Their layered structures facilitate the conversion of polyolefins into hydrocarbon-rich liquids while limiting coke and wax formation. For instance, Liu et al. [37] reported complete conversion of polyolefins at 430 °C using MMT as a catalyst, which yielded the highest proportion of liquid products (oil + wax, 16%), predominantly alkanes, when compared with other catalysts such as HZSM-5, MCM-41, Al_2_O_3_, and CaO. The superior performance of MMT was attributed to its capacity for intermolecular hydrogen transfer and inhibition of β-scission via weak Lewis acid sites and large mesopores. Similarly, Budsaereechai et al. [110] demonstrated that bentonite-assisted pyrolysis of mixed plastics (PS, PP, PE) in a bench-scale reactor achieved over 88% liquid hydrocarbon yields within the gasoline-diesel range, with negligible wax or char formation. Bentonite enhanced polymer cracking and condensable gas generation, thereby improving liquid oil yields and suppressing heavy wax (C_13_–C_28_) formation. The relatively mild acid strength of pristine clays supports β-scission and radical stabilization, yielding diesel-range aliphatic hydrocarbons from LDPE, PP, and HDPE, while PS primarily generates gasoline-range aromatic compounds.

Acid activation further enhances the catalytic performance of MMTs by introducing stronger acid sites and generating mesopores that suppress wax formation due to chain scission and promote the formation of C_5_–C_11_ gasoline range hydrocarbons. Under optimized conditions, oil yields approach 90% [103,111]. K10, a representative acid-activated MMT, demonstrates remarkable deoxygenation efficiency. Rutkowski et al. [103] reported that K10 reduced the oxygen content of the fuel oil derived from beverage carton waste from 22.4% to 11.0%, due to its strong acid sites that catalyze dehydration and deoxygenation. In comparison, non-catalytic pyrolysis led to wax formation and phase-separated pyrolytic liquids. K10 also exhibited higher dehydrogenation activity, yielding paraffinic-olefinic mixtures, while KSF (its characteristics in Table 3) produced slightly higher liquid yields but with lower deoxygenation and aromatic content. Subsequent work by Rutkowski [40] on co-pyrolysis of cellulose and polyethylene (see Section 1 for co-pyrolysis mechanism) confirmed that K10 enhanced the dehydration and deoxygenation of anhydrosugars (levoglucosan, AGF), leading to the highest share of liquid hydrocarbons and more deoxygenated oils, albeit with increased gas yield. More recently, Cai et al. [111], reported that K10 in mixed-plastic systems slowed secondary decomposition of light oils into gaseous products, thereby maximizing the accumulation of light hydrocarbons. Compared to pristine systems, acid-activated MMTs exhibit more balanced cracking behavior, producing well-distributed hydrocarbon fractions while maintaining low coke deposition, an essential advantage for continuous pyrolysis reactors.

De Stefanis et al. [41] synthesized pillared clays from acid-activated K10 and evaluated them for plastic degradation. As expected, K10 primarily yielded aliphatic hydrocarbons, whereas HZSM-5 favored aromatics, gases, and light olefins. In contrast, Fe/Al-PILC promoted gasoil-range hydrocarbon formation (C_12_–C_23_) and achieved the highest fuel oil yield (65%) compared to non-catalytic pyrolysis, attributed to improved hydrogen-transfer activity. Faillace et al. [39] further demonstrated that Fe-PILC derived from purified MMT selectively cracked a heavy gas oil (HGO) and polyolefin mixture into light hydrocarbons (C_10_–C_23_) suitable for diesel-range fuels. The production of linear hydrocarbons, rather than aromatics, was attributed to the larger mesopore volume of the pillared structure.

More complex catalytic systems have emerged through transition-metal doping of PILCs (e.g., Al^3+^, Fe^2+^, Zn^2+^,Ti^4+^, Ni^2+^/Co^2+^), offering superior performance in mixed-plastic pyrolysis [112,113,114]. The expanded interlayer spacing and enhanced acidity of these materials facilitate the diffusion of bulky polymer chains and stabilize intermediate radicals during cracking. Under optimized conditions, overall liquid yields exceed 80%. Fe-PILC exhibits high selectivity toward C_13_–C_19_ diesel range hydrocarbons, reduced C_20_–C_40_ fractions, and significant H_2_ evolution, consistent with its moderate acidity. Conversely, Al-PILC favors light hydrocarbon and aromatic formation, producing benzene and naphthalene via cyclization and aromatic ring cleavage from PS and PET [112]. The generation of heavy polycyclic aromatics has been associated with Brønsted acid driven aromatization and Lewis acid induced dehydrogenation pathways. Upscaling studies in stirred tank reactors confirmed that Fe-PILC promoted C_9_–C_19_ fraction formation (73.38%) at 450 °C compared to non-catalytic systems that yielded a broader C_6_–C_30_ distribution [113]. To further enhance oil quality and product selectivity, Li et al. [114] developed Ni/Co-PILC catalysts, achieving the highest yield of light liquids (<C_9_, 27.2%) due to their favorable textural structure and moderate acidity, which effectively suppress over-cracking. Notably, cycloparaffins dominated the liquid fraction (up to 31%), a result of cyclization and alkylation reactions facilitated by the Lewis acidity of Co/Ni-PILC. These systems outperform pristine and acid-activated clays in both product selectivity and hydrocarbon quality. The incorporation of transition metal oxides within the MMT framework not only enhances catalytic longevity and resistance to coking but also renders these materials promising candidates for industrial-scale polymer waste valorization. Nevertheless, as in biomass pyrolysis, the absence of reducing environments restricts these catalysts to acid-driven mechanisms, necessitating subsequent hydrogenation and hytroatome (oxygen) removal for complete fuel upgrading.

#### 3.3.3. Impact of Catalyst Modification on Biofuel and RCF Production

**Figure 11:**
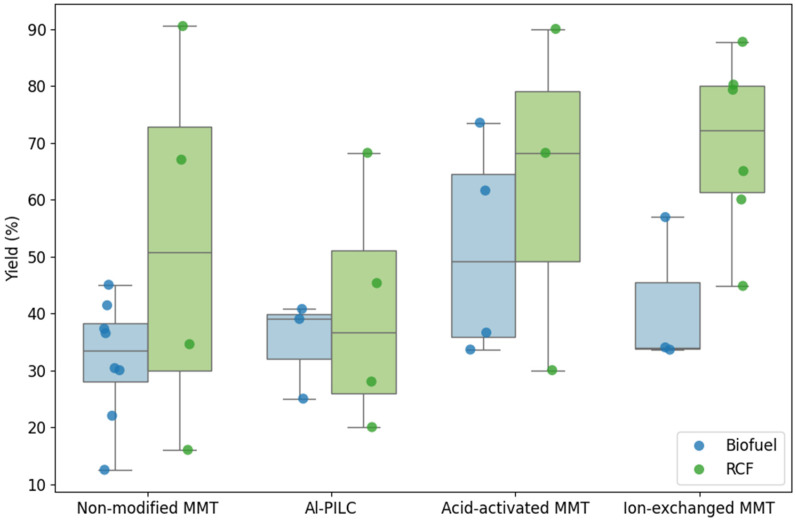
Distribution of upgraded oil yields for biofuel and RCF using different MMT-based catalysts.

Building on the previous sections that described the applications of MMT-based catalysts in biomass and plastic pyrolysis, this subsection examines how different MMT modifications influence upgraded oil yields. The analysis considers both feedstock types—biofuel and RCF—to evaluate the effect of catalyst structure on both process efficiency and reproducibility. Data from 31 studies (see Table S1, Supplementary Materials) were visualized using box and whisker plots (see Figure 11), allowing for a comparative assessment of non-modified, ion-exchanged, acid-activated, and pillared MMT catalysts.

The results demonstrate that catalyst type strongly affects both the magnitude and variability of yields. For biofuel production, non-modified MMT yielded moderate oil conversion with a relatively narrow interquartile range (IQR = 10.3%), indicating consistent performance but limited enhancement. In contrast, RCF conversion with the same catalyst achieved higher median yields, yet displayed considerably larger variability (IQR = 42.96%), suggesting less predictable outcomes under similar conditions. Al-PILC and acid-activated MMT showed analogous trends: biofuel yields were consistently lower than those for RCF, while their smaller IQRs indicate better reproducibility for biofuel applications.

Ion-exchanged MMT emerged as the most effective catalyst for RCF production, attaining the highest median yield (72.15%) with moderate variability (IQR = 18.73%). For biofuel, its performance was lower, ranging between 34% and 45.45%, highlighting the influence of feedstock–catalyst interactions on product distribution. Overall, RCF yields were higher but more scattered, whereas biofuel yields were more homogeneous across catalyst types. This contrast underscores the importance of matching catalyst modification to the desired process objective: reproducibility for biofuel versus maximum yield for RCF.

These observations have direct implications for marine oil applications. Non-modified, Al-PILC, and acid-activated MMT are preferable when process consistency and predictable biofuel quality are critical. Conversely, ion-exchanged MMT is recommended when prioritizing high RCF yield, albeit with greater variability. The analysis thus emphasizes that the choice of catalyst modification should be guided by a balance between yield optimization and reproducibility, tailored to the specific feedstock and end-use requirements.

### 3.4. Hydrodeoxygenation over MMT-Based Catalysts

Following catalytic cracking and pyrolysis, the resulting bio-oils still contain a high proportion of oxygenated compounds, hindering their direct utilization as transportation fuels. Hydrodeoxygenation therefore represents a crucial upgrading step, in which reactive oxygenates are converted into fuel-range hydrocarbons through hydrogen-assisted deoxygenation reactions. While MMT acts mainly as a solid acid catalyst promoting depolymerization and dehydration during pyrolysis, its role in HDO extends to functioning as a hybrid acid-metal catalyst. In such systems, finely dispersed transition metals (e.g., Ni^2+^_,_ Co^2+^, Ru^3+^, Pd^2+^, Mo^4+^) supported on MMT facilitate both C-O bond scission and hydrogen addition through a synergistic acid-metal mechanism.

Pristine MMT exhibits moderate Lewis and Brønsted acidity but lacks metallic hydrogenation sites, resulting in limited intrinsic HDO activity. For instance, during the hydroxyalkylation of p-cresol using formaldehyde as a solvent, MMT showed negligible catalytic activity [115]. To overcome this limitation, metals such as Al^3+^, Zn^2+^, and Fe^3+^ have been introduced into the MMT framework. Among these, Al-MMT demonstrated remarkable recyclability and maintained high catalytic performance even after eight consecutive reaction cycles, showing no loss in selectivity (98%). Similar observations were reported for the HDO of various phenolic compounds (phenol, 4-propylphenol, 1,4-benzenediol, anisole, and diphenyl ether), where immobilizing Ru nanoparticles within an ionic-liquid-modified MMT promoted the efficient deoxygenation to the corresponding cycloalkanes [116]. The ionic liquid layer enhanced metal dispersion and accessibility, fostered acid-metal synergy, and prevented catalyst deactivation by agglomeration or coking. The resulting Ru-MMT catalyst achieved complete conversion and 100% selectivity toward cycloalkanes, with excellent stability due to the absence of Ru leaching in aqueous media. Comparable efficiency has been reported for Ni-supported MMT catalysts. Hengne et al. [117] demonstrated that Ni-MMT exhibited exceptional activity for the conversion of levulinic acid (LA) to γ-valerolactone (GVL) under transfer hydrogenation conditions using isopropanol as a hydrogen donor. Nearly full conversion and >99% selectivity was achieved. The strong acidity of the clay facilitated the esterification of LA with isopropanol and the subsequent cyclization to GVL, while in situ reduction of Ni^2+^ to Ni_0_ provided active metallic sites. Furthermore, Fe/Ni-MMT bimetallic systems enhanced this reactivity through improved redox balance and hydrogen transfer efficiency, addressing persistent challenges in HDO such as metal dispersion loss and deactivation by polymeric residues.

**Figure 12:**
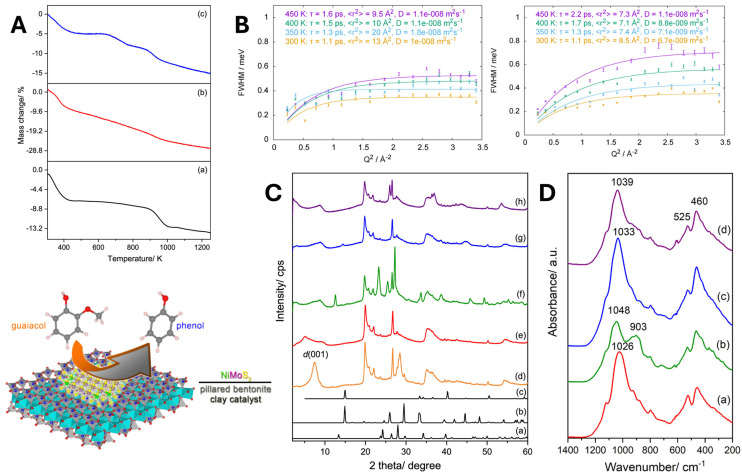
(**A**) TGA profile of: (**a**) MMT; (**b**) Al-PILC; (**c**) Ni/Mo/Al-PILC (sulfided). (**B**) adsorption of guaiacol using QENS measurements and data fitted using Lorentzian peak fit, dosed on Al-PILC on the left and Ni/Mo/Al-PILC (sulfided) on the right. (**C**) XRD patterns of: (a) MoO_3_, (b) α-NiMoO_4_, (c) MoS_2_, (d) MMT, (e) Al-PILC, (f) Ni/Mo/Al-PILC (g) Ni/Mo/Al-PILC (reduced), (h) Ni/Mo/Al-PILC (sulfided). (**D**) IR spectra of catalysts: (a) Al-PILC; (b) Ni/Mo/Al-PILC; (c) Ni/Mo/Al-PILC (reduced), (d) Ni/Mo/Al-PILC (sulfided). Reproduced from ref. [121], Copyright © 2019 by Indri B. Adilina is licensed under CC BY.

Kasar et al. [118] prepared a Ru/Ni-K10 catalyst via the impregnation-precipitation method using acid-activated clay. Although the conversion was slightly lower than in previous systems, the result was attributed to a shorter reaction time and the accumulation of NiO species, which promoted pentanoic acid formation. In contrast, Pd-K10 catalysts showed remarkable performance in the conversion of molecular bio-oil models. According to X. Wang et al. [119], phenol did not undergo direct hydrogenolysis to benzene, and guaiacol conversion at elevated temperatures yielded modest cycloalkane amounts, constrained by the presence of methoxy groups. These moieties represent kinetic bottlenecks that require cooperative demethoxylation-hydrogenation over adjacent acid and metal sites. Pd-K10 effectively depolymerized lignin-derived model compounds, achieving nearly complete deoxygenation and high carbon retention while promoting subsequent hydrogenation to cycloalkanes. Consistent trends were observed in more complex bio-oil model conversions, such as isovanillin, vanillin, benzophenone, and 3,4-dimethoxybenzaldehyde, using Ni-K10 catalysts [120]. Additionally, the sulfided Ni/Mo/Al-PILC system exhibited superior HDO performance. In the degradation of guaiacol, this catalyst achieved complete conversion (100%) to partially deoxygenated products, primarily phenol (77%) and o-cresol (22%), at 350 °C and 20 bar H_2_ [121]. The results highlight the cooperative action of acidic sites for demethoxylation and metallic sites for hydrogenation, enhanced by the open framework and diffusion properties of the pillared structure. Complete oxygen removal (C = 100%) was achieved (see Figure 12), confirming that the acid–metal interplay is indispensable for efficient HDO performance. Additional hydrodeoxygenation data for related catalytic systems [122,123,124,125,126,127,128,129], including reaction conditions and liquid product conversion and selectivity, are summarized in Table S1, Supplementary Materials.

## 4. Research Gaps and Future Perspectives

To the best of our knowledge, no comprehensive review has yet addressed the complete upgrading chain of real bio-oil systems using MMT-based catalysts, spanning feedstock pyrolysis and co-pyrolysis, catalytic hydrotreatment, and final fuel-quality evaluation. The existing literature remains largely confined to model compounds or simplified systems, leaving a substantial gap between laboratory-scale investigations and process-level applications. Bridging this gap is essential to assess the true potential of MMT and its modified derivatives as cost-effective catalysts for sustainable alternative fuel production. This research limitation may stem from the fact that most upgrading experiments are still conducted on a small scale, where insufficient quantities of bio-oil hinder testing according to established fuel standards, such as those applicable to marine biofuels.

Future research should prioritize the application of MMT-based catalysts to realistic and chemically complex bio-oil feeds characterized by high oxygen content, significant water fractions, and polymeric residues, in order to assess catalyst robustness under practical conditions. Particular attention should be devoted to mitigating carbon deposition through targeted strategies such as acidity tuning, surface modification, and controlled dispersion or anchoring of active metal species. In parallel, systematic studies under hydrothermal and reactive environments are required to elucidate metal leaching mechanisms and to identify structural features of MMT that enhance metal–support interactions. Long-term stability tests, including multiple reaction–regeneration cycles, should be conducted to evaluate coking resistance, regeneration efficiency, and structural integrity. These investigations are essential to determine whether optimized clay-based catalysts can achieve stable performance levels comparable to conventional zeolites in catalytic upgrading processes.

Another critical aspect concerns the analytical characterization of upgraded bio-oils. Most studies rely predominantly on GC-MS analysis, which provides information limited to the volatile, low-molecular-weight fraction while neglecting the heavier, polymeric, and oxygen-rich species that significantly influence fuel quality. A more comprehensive approach combining techniques such as SEC/GPC, GC×GC-MS, FTIR, NMR, and elemental analyses is necessary to capture the full molecular distribution of upgraded products and to assess their compatibility with established marine fuel standards.

Furthermore, future work should incorporate techno-economic and life cycle analyses (TEA/LCA) that account for catalyst synthesis, metal costs, regeneration frequency, and hydrogen demand. Such evaluations are crucial for determining the long-term viability and sustainability of MMT-based catalysts relative to zeolitic and sulfided NiMo systems. Pilot-scale reactor studies will also be essential to validate laboratory findings, providing insight into operational challenges such as mass-transfer limitations, coking dynamics, and thermal management.

Finally, promising advances are expected from the development of hydrogen-free upgrading routes, such as solvolysis, which could substantially reduce the reliance on external hydrogen. To date, however, no studies have investigated the application of MMT-based catalysts in this context. Exploring this approach offers a particularly promising opportunity, as the tunable acidity and layered structure of MMT could facilitate selective deoxygenation, limit polymerization and coking, and enhance catalyst stability. Such studies could also expand the range of bio-oil feedstocks that can be effectively upgraded and provide insights into scalable, sustainable alternatives to conventional hydrogen-dependent processes.

## 5. Conclusions

This review provides a comprehensive and integrated assessment of MMT-based catalysts for the production of alternative marine fuel feedstocks, encompassing the entire conversion chain from feedstock pyrolysis to catalytic upgrading and the qualitative evaluation of product distribution trends. By jointly examining lignocellulosic biomass derived bio-oils and polymer-derived recycled carbon fuels, the work offers a unified perspective that is largely absent from existing reviews, which typically focus on isolated upgrading steps or on zeolitic catalysts alone. Compared with conventional zeolites, MMT-based catalysts exhibit several intrinsic advantages. Their natural abundance, low raw-material cost, and environmentally benign nature make them attractive from both economic and sustainability perspectives. In addition, their layered structure, tunable acidity, high ion-exchange capacity, and ability to disperse active metal species provide significant flexibility in catalyst design. These characteristics translate into enhanced tolerance toward oxygen-rich, water-containing, and chemically complex feeds, as well as reduced susceptibility to severe coking under mild catalytic cracking and hydroprocessing conditions. In contrast, while zeolites offer strong Brønsted acidity and high deoxygenation severity, they often suffer from rapid deactivation, limited feedstock flexibility, and higher material costs.

The systematic synthesis of the literature further highlights that catalyst modification plays a decisive role in steering product distribution. Ion-exchanged MMT emerges as the most effective option for RCF production, achieving the highest median oil yields, albeit with greater variability. In contrast, biofuel yields from lignocellulosic feeds are more homogeneous across catalyst types, with non-modified, acid-activated, and Al-pillared MMT offering improved reproducibility. These findings emphasize that catalyst selection should be guided by process objectives: maximizing yield in RCF-oriented pathways versus ensuring consistency and predictability in biofuel production. Such considerations are particularly relevant for marine fuel applications, where blending strategies and fuel-grade requirements demand stable and controllable product qualities.

Despite these advantages, several critical challenges remain. Deep deoxygenation to levels compatible with stringent marine fuel standards still often requires hydrogen-intensive hydrodeoxygenation, and the long-term stability of MMT-based catalysts under realistic upgrading conditions remains insufficiently explored. Most existing studies rely on molecular model compounds or short-duration experiments, leaving a substantial gap between laboratory-scale demonstrations and process-level validation using real bio-oil systems. Issues related to carbon deposition, metal leaching, regeneration efficiency, and hydrothermal stability must therefore be addressed through systematic long-term testing, including multiple reaction–regeneration cycles. To the best of our knowledge, no prior review has addressed the complete upgrading chain of real bio-oil systems using MMT-based catalysts, spanning feedstock pyrolysis and co-pyrolysis, catalytic hydrotreatment, and fuel-quality considerations. By identifying these gaps and consolidating current knowledge, this review provides a structured framework for evaluating MMT-based catalysts as flexible, cost-efficient, and potentially robust alternatives to zeolites. Future research should prioritize realistic feedstocks, integrated process testing, and techno-economic and fuel-standard assessments to determine whether optimized clay-based catalysts can achieve stable performance levels comparable to conventional systems and contribute meaningfully to the decarbonization of marine transport.

## Data Availability

No new data were created or analyzed in this study. Data sharing is not applicable to this article.

## Supplementary Materials

The following supporting information can be downloaded at: https://www.mdpi.com/article/10.3390/molecules31020339/s1, Table S1: Overview of the literature on organic conversions of biomass and plastics to alternative fuels catalyzed by pristine, ion-exchanged, acid activated, and pillared montmorillonite.
