# Supplementary material for: A Review on Modified Montmorillonite-Based Catalysts for Biofuel and Recycled Carbon Fuel Production
Source: Molecules. 2026 Jan 19;31(2):339. doi: 10.3390/molecules31020339 (PMC12843678; doi:10.3390/molecules31020339)
Supplement: Supplementary file 1 [file molecules-31-00339-s001.zip › molecules-4043357-supplementary.pdf]

Table S1. Overview of the literature on organic conversions of biomass and plastics to alternative fuels catalyzed by pristine, ion-exchanged, acid activated, and pillared montmorillonite.

Feedstock	Catalyst Type	Preparation Method	Reactor	Temperature (°C)	Pressure	Catalyst Loading (wt%)	Time (min)	Liquid Product (Y/C/S (%)	Ref
**Catalytic Cracking**									
"Tectona Grandis" Wood chip pyrolysis Bio-oil	MMT<br>Al-PILC	Commercial<br>Al_{13} Keggin ion synthesis, ion exchanged with MMT and calcined	Fixed bed	300-500	1 bar	N. D	N. D	Al-PILC: Y=25% (at 300°C)	[109]
Bamboo straw, Corncob, Corn Straw, Sawdust, or Wheat straw	K10<br>Fe-K10	Commercial<br>Pyrolyzing Coordinated Polymer (PCP) strategy where both Fe and supporting ligand, were supported on MMT	Fixed bed	500	N_2=50 ml/min	10%	30	Fe-K10: Y=56.9% (corncob)	[105]
Pine Sawdust	K10<br>Fe-K10	Commercial<br>Wet-impregnation on MMT with Fe loading: 0%, 5% and 10%	Dual reactor: pyrolysis and ex-situ upgrading	600	N_2=1250 ml/min	30.33-40-50%	15	Fe-K10: Y=33.60% (5% Fe loading)<br>Fe-K10: Y=31% (10% Fe loading)	[104]
"Saccharina Japonica", Kelp	Bentonite (B)	Commercial	Fixed bed	600	N_2=200 ml/min	5-10-20%	60	B: Y=36.5% (20% loading)	[102]
Corn stalk	K10<br>Co-K10	Commercial<br>Cobalt exchanged MMT at different cobalt salt amounts: 0,3-0,6-0,9-1,2g	Thermogravimetric analysis (TGA)<br>Fixed bed	30-700<br>500	N_2=50 ml/min<br>Ar=70 ml/min	5-10-15-20%	67	K10: Y=33.62% (20% loading)<br>Co-MMT (0.6g Co-loading): Y=34% (15% loading)	[107]

Almond shell pyrolysis Bio-oil	Bentonite (B)	Commercial	Fixed bed	400	N. D	30%	30	B: Y=76.38% (representing 37.27% based on almond shell mass)	[97]
"Pinus halepensis", Forest Pine woodchips	Bentonite (B) plus sand	Commercial, with sand + B to biomass weight ratio = 3:1	Auger Bed	450	N. D	33%	N. D	B: Y=45%	[101]
Paper mill waste (PMW)	MMT	Commercial	Thermogravimetric analysis (TGA)	30-1000	N. D	50%	Depending on heating rates: 20-25-30 °C/min	MMT: Y=82.72% (liquid and gas yields)	[122]
Cherry Pits (CP)	MMT	Commercial	Horizontal Fixed Bed tube furnace	600	N_2=100 ml/min	10%	60	MMT: Y=61.57%	[98]
Safflower seeds pyrolysis Bio-oil	K10<br>Al-K10	Commercial<br>Aluminum ion exchanged MMT at 10% Al loading	Fixed bed	300-600	N_2=100 ml/min	N. D	N. D	Al-K10: Y=40.75% (at 550°C)	[106]
"Eriophorum Sphagnum", Peat	Bentonite (B)	Commercial	Fixed bed	460	N. D	30%	N. D	B: Y=30.36%	[99]
Avocado pits	Bentonite (B)	Commercial	Porcelain boat<br>Batch reactor	600<br>250	N_2=100 ml/min<br>40 bar	20%<br>20%	30<br>60	B: Y=40.45<br>B: Y=41.40	[100]
Pine sawdust	MMT	Commercial	Fixed bed	500	N_2=1200 ml/min	20%	30	MMT: Y around 30%	[123]
Canola oil	Al-PILC	Al_{13} Keggin ion synthesis, ion exchanged with MMT and calcined	Fixed bed microreactor	375-500	Ar=16 ml/min	N. D	30	Al-PILC: C=87.60% (at 550°C, 1,8 whsv.h^{-1}), Y=39%	[108]
Alkaline Lignin	Bentonite (B)<br>H-MMT	Commercial<br>Acid activated MMT with HCl, H_2SO_4, H_3PO_4	Down-flow fixed-bed quartz	500-650	N_2=25-100 ml/min	20-25-33-50%	N. D	H-MMT (HCl): S(BTX)=60% (at 600°C, N_2=75 ml/min, 25wt%)	[124]

High Density Polyethylene (HDPE)	MMT	Commercial	Thermogravimetric analysis (TGA)	30-900	N_2=100 ml/min	10%	60	MMT: Y=16%	[37]
			Quartz boat	430	Ar=270 ml/min				
Low-Density Polyethylene (LDPE), Mix plastics: LDPE: HDPE: PP (41: 24: 35)	MMT	Commercial	Thermogravimetric analysis (TGA)	30-600	N_2= 100 ml/min	50%	N. D	Al-PILC: Y=45.3% (Batch reactor, LDPE)	[38]
	Al-PILC	Commercial	Double-shot pyrolizer coupled to GC-MS	450-650	He=1 ml/min			Al-PILC: Y=44.8% (Batch reactor, Mixture plastics)	
	Co/H-MMT, Mo/H-MMT, Ni/H-MMT, Zn/H-MMT	Acid modified MMT, and ion exchanged with (Co, Mo, Ni, Zn)	Batch reactor	500	N. D				
HDPE, Heavy Gas oil (HGO), Mixture	ARG	Sodium activated bentonite treated with iron removal	Tubular reactor	450-500	N_2=10 ml/min	10%	N. D	Al-PILC450: Y=around 28% (at 450°C)	[39]
	Al-PILC450	Al_{13} Keggin ion synthesis, ion exchanged with ARG and calcined at 450						Al-PILC-Fe450: Y=around 50% (at 450°C)	
	Fe-PILC-Fe450	Fe pillaring solution, ion exchanged with original clay ARG before Fe removal and calcined at 450						Fe-PILC-Fe450: Y=around 60% (at 450°C)	

Cellulose and Low-Density Polyethylene (CPE) mixture	K10<br>KSF<br>Bentonite (B)	Commercial	Horizontal quartz reactor	400-500	N_2=167 ml/min	50%	Fast heating 100°C/s plus 5 min	K10: Y=70% (at 500°C)<br>B: Y=64% (at 500°C)<br>KSF: Y=52% (at 500°C)	[40]
Medium-Density Polyethylene (MDPE)	K10	Commercial	Fixed bed and 10 mL Pyrex	300	N. D	50%	240	K10: Y=around 30%	[41]
	H-MMT	Acid-treated restricted MMT obtained through milder acid restructing						H-MMT: Y=around 60%	
	Al-PILC	Al13 Keggin ion synthesis, ion exchanged with restricted MMT and calcined						Al-PILC: Y=around 20%	
	Fe/Al-PILC	Double ion exchanged Al oxide and Fe oxide in second phase with restricted MMT						Fe/Al-PILC: Y=around 65%	
Mixed plastics waste (PE: PP: PS: PET=40: 20: 20: 20)	K10	Commercial	Fixed bed	500	N_2=50 ml/min	10%	60	K10: Y=around 90% oil with 30% wax	[111]
PP, LDPE, HDPE, PS	Bentonite (B)	Pellets are formed on compression of the catalyst powder with hydraulic press at pressure of 50 bar	Bench scale fixed bed	500	N. D	4.8-9.1-13-16.7%	10	B: Y=88.5% (PS at 4.8%)<br>B: Y=90.5% (PP at 9.1%)<br>B: Y=87.6 (LDPE at 16.7%)<br>B: Y=88.9 (HDPE at 13%)	[110]
Mixed plastic wastes (HDPE: PP: PS: PET 42: 35: 18: 5)	Al-PILC	Intercalation of Al_{13} Keggin ion with Bentonite, and calcined	Horizontal fixed bed	500	N_2=200 ml/min	10%	30	Al-PILC: Y=68.2	[112]
	Fe-PILC<br>Zr-PILC<br>Ti-PILC	Intercalation using different polynuclear metal oxo-hydroxo cations						Fe-PILC: Y=79.3<br>Ti-PILC: Y=62.8<br>Zr-PILC: Y=62.1	
	Fe-RC	Iron restructured clay	Stirred tank	425-500	N_2=1000 ml/min	1.79-3.51-5.17-6.78%	10	Fe-RC: Y=78.37% (at 425°C, 1.79%)	[113]

Mixed plastic wastes (PE: PP: PS: PVC: PET= 42: 35: 16: 5: 2)								Fe-RC: Y=75.27 (at 450°C, 1.79%)<br>Fe-RC: Y=83.73% (at 450°C, 3.51%)	
Film waste	Na-MMT<br>O-MMT<br>Co-PILC<br>No-PILC<br>Co/Ni-PILC	Naturel MMT saturated with NaCl<br>Na-MMT, treated with hexadecyl-trimethyl ammonium bromide by ion exchange (O-MMT)<br>Intercalation using metal chelate cations (Co, Ni, and ligand), and calcined	Tubular furnace	540	N. D	10%	30	Na-MMT: Y=67%<br>O-MMT: Y=68.6%<br>Co-PILC: Y=80<br>Ni-PILC: Y=76<br>Co/No-PILC Y=80.2	[114]
Beverage carton waste: tetra aseptic (TA), cellulose (TAC), polyethylene (TAPE)	K10<br>KSF	Commercial<br>Commercial	Quartz reactor	450	N_2=167 ml/min	33-50-67%	Fast heating 100°C/s plus 5 min	KSF: Y=69.3% (TA at 50%)<br>K10: Y=61% (TA at 67%)<br>K10: Y=63.5% (TA at 50%)<br>K10: Y=49.7% (TA at 33%)	[103]
Waste Polypropylene	MMT	Commercial	Batch reactor	500	N_2=50 ml/min	5%	180	MMT: Y=34.56%	[125]
Sewage Sludge (SS)	MMT	Commercial	Fixed bed	400-800	Ar=50 ml/min	40%	60	MMT: Y_N = 14.14% (compared to 28.7% in non-catalytic)	[126]
Oily sludge	Bentonite (B)<br>Fe/Al-PILC	Commercial<br>Double ion exchanged Al oxide and Fe oxide in second phase with Bentonite	Horizontal quartz reactor	450	N_2=100 ml/min	2%	180	Fe/Al-PILC: Y= around 52%	[127]

Cellulose	K10	Commercial	Quartz phial fixed on a stainless-steel flange	350-500	N_2=25 ml/min	50%	5	K10: Y=17.8 (of LAC at 500 °C)	[128]
Cellulose	K10	Commercial	Fixed bed	300-500	N_2=167 ml/min	10-25- 50%	Fast heating 100°C/s, and slow heating 10°C/min	K10: Y=73.5% (at 450°C, 100°C/s, 10%)<br>K10: Y=71.2% (at 450°C, 100°C/s, 25%)<br>K10: Y=64.4% (at 450°C, 100°C/s, 50%)<br>K10: Y=68% (at 500°C, 100°C/s, 50%)	[129]
				**Hydrodeoxygenation**					
Guaiacol	Bentonite (B)<br>Ni/Mo/Al-PILC (reduced)<br><br>Ni/Mo/Al-PILC (sulfide)	Commercial<br>Al_{13} Keggin ion synthesis, ion exchanged with MMT, then double impregnation with Ni and Mo, then reduced or sulfide	High Pressure Autoclave	350-450	H_2=2.5-20 bar	5%	360	Al-PILC: C=78%<br>Ni/Mo/Al-PILC (reduced) C=73%<br><br>Ni/Mo/Al-PILC (sulfide) C=100%	[121]
p-Cresol	MMT<br>Al-MMT<br>Zn-MMT<br><br>Fe-MMT	Commercial<br><br>MMT ion exchanged with Al, Zn, Fe	Round bottom glass	80	p-cresol/Formaldehyde mole ratio 3:1	1.36-1.96%	120	Al-MMT: C=51%, S=98% (to DAM)	[115]
Vanillin, and various biomass models: a) Isovanillin, b) 3-4-Dimethoxy-Benzaldehyde, c) Syringaldehyde, d)	K10<br><br>Ni-K10	Commercial<br><br>10% Ni supported on K10	Autoclave	180	Isopropanol, with N_2= 10 bar	40%	180<br><br>120	Ni-K10: C=99%, S=95.2, Y=95.3 (to 2-methosy-4 methyl phenol)<br>Various biomass model on Ni-K10: a) C=98.2%, S=94.7, Y=92.9 (to 2-	[120]

p-Anisaldehyde, e) Benzaldehyde, f) Cinnamaldehyde, g) Benzophenone								methoxsy-3 methyl phenol)<br>b) C=100, S=95, Y=95 (to 1,2-dimethoxy-4-methylbenzene)<br>c) C=95.8, S=96.2, Y=91.9 (to 4-Methyl-2,6-dimethoxyphenol)<br>d) C=98.1, S=97.5, Y=95.6 (to 1-methoxy-4-methylbenzene)<br>e) C=82.6, S=85.4, Y=70.5 (to methylbenzene)<br>f) C=98.6, S=43.7, Y=43 (to phenyl-4 propanol)<br>g) C=74.5, S=62.8, Y=46.7 (to diphenylmethane)	
Phenol and various lignin-derived phenolic compounds: a) Guaiacols, b) Phenethoxy-benzene (55% in lignin), c) Benzyloxy-benzene (8% in lignin), d) Diphenyl ether	Na-MMT<br>H-MMT<br>Pd/Na-MMT<br>Pd/-MMT	Commercial<br>Acid activated from Na-MMT<br>Na-MMT ion exchanged with palladium nitrate, and calcined<br>H-MMT ion exchanged with palladium nitrate, and calcined	Autoclave	200	3ml H_2O, H_2=50 bar	21%	120-480	Phenol: Na-MMT: C=0, Y=0<br>Phenol: Pd/Na-MMT: C=100, Y=56 (to cyclohexanone)<br>Phenol: Pd/H-MMT: C=100, Y=99 (to cyclohexane)<br>Various lignin on Pd/H-MMT:<br>a) C=100, Y= 50%-73% (dependent on mono or di-guaiacol), b) C=100, Y=18% (to cyclohexane), Y=53% (to ethyl-	[119]

								cyclohexane), c) C=100, Y= 29% (to cyclohexane), Y=46% (to methyl-cyclohexane), d) C=100%, Y=85% (to cyclohexane)	
Levulinic acid	Ru/Ni-K10	0.5%Ru, 5%Ni supported on K10, were prepared by wet impregnation-precipitation	Autoclave	160-240	95 ml H_2O, H_2=17.2 bar	6%	300	Ru/Ni-K10: C=91% (at 220°C), S=100% (to GVL)	[119]
Levulinic acid	Ni-MMT	Ni ion exchanged with MMT	Autoclave	200	N_2=atm	50%	60-150	Ni-MMT: C=99%, S=99% (to GVL at isopropanol H-donor). C=99%, S=98% (to Levulinic esters at ethanol H-donor)	[117]
a) Phenol, b) 4-propylphenol, c) 1,4, benzene diol, d) Phenol, methoxy, e) Di-phenyl ether	Na-MMT<br><br>Ru-MMT @ILSO$_3$H	Commercial<br><br>MMT modified with functional ionic liquid (@ILSO$_3$H), and redispersed with Ru nanoparticles onto the MMT	Autoclave	200	3 ml H_2O, H_2=50 bar	15%	60-120	Ru-MMT@ILSO$_3$H at 60 min: a) S=100 (to cycloalkanes, compared to S=100 to cycloalcohol with Ru/MMT), b) S=86 (to cycloalkanes)<br>Ru-MMT@ILSO$_3$H at 120 min: b) S=100% (to cycloalkanes), c) S=98% (to cycloalkanes), d) S=72% (to cycloalkanes), e) S=97% (to cycloalkanes)	[116]

References

37. Liu, M.; Zhuo, J.K.; Xiong, S.J.; Yao, Q. Catalytic Degradation of HDPE over Clay Catalysts. Energy Fuels 2014, 28, 6038–6045. https://doi.org/10.1021/ef501326k.
38. Dai, L.; Zhou, N.; Lv, Y.; Cheng, Y.; Wang, Y.; Liu, Y.; Cobb, K.; Chen, P.; Lei, H.; Ruan, R. Chemical Upcycling of Waste Polyolefins. Sci. Total Environ. 2021, 782, 146897. https://doi.org/10.1016/j.scitotenv.2021.146897.
39. Faillace, J.G.; De Melo, C.F.; De Souza, S.P.L.; Marques, M.R.C. Catalytic Pyrolysis of HDPE Using Pillared Clays. J. Anal. Appl. Pyrolysis 2017, 126, 70–76. https://doi.org/10.1016/j.jaap.2017.06.023.
40. Solak, A.; Rutkowski, P. Clay Catalyst Effects on Bio-Oil Composition. Waste Manag. 2014, 34, 504–512. https://doi.org/10.1016/j.wasman.2013.10.036.
41. De Stefanis, A.; Cafarelli, P.; Gallese, F.; Borsella, E.; Nana, A.; Perez, G. Catalytic Pyrolysis of Polyethylene. J. Anal. Appl. Pyrolysis 2013, 104, 479–484. https://doi.org/10.1016/j.jaap.2013.05.023.
97. Kar, Y. Catalytic cracking of pyrolytic oil using bentonite clay for green liquid hydrocarbon fuel production. Biomass Bioenergy 2018, 119, 473–479. https://doi.org/10.1016/j.biombioe.2018.10.014.
98. Karod, M.; Hubble, A.H.; Maag, A.R.; Pollard, Z.A.; Goldfarb, J.L. Clay-catalyzed in situ pyrolysis of cherry pits for upgraded biofuels. Biomass Convers. Biorefin. 2024, 14, 7873–7885. https://doi.org/10.1007/s13399-022-02921-3.
99. Sulman, M.; Kosivtsov, Y.; Sulman, E.; Alfyorov, V.; Lugovoy, Y.; Molchanov, V.; Tyamina, I.; Misnikov, O.; Afanasjev, A.; Kumar, N.; et al. Influence of aluminosilicate materials on peat low-temperature pyrolysis. Chem. Eng. J. 2009, 154, 355–360. https://doi.org/10.1016/j.cej.2009.04.001.
100. Karod, M.; Pollard, Z.A.; Ahmad, M.T.; Dou, G.; Gao, L.; Goldfarb, J.L. Impact of bentonite clay on in situ pyrolysis vs. hydrothermal carbonization of avocado pit biomass. Catalysts 2022, 12, 655. https://doi.org/10.3390/catal12060655.
101. Veses, A.; Aznar, M.; López, J.M.; Callén, M.S.; Murillo, R.; García, T. Production of upgraded bio-oils by biomass catalytic pyrolysis. Fuel 2015, 141, 17–22. https://doi.org/10.1016/j.fuel.2014.10.044.
102. Sewu, D.D.; Lee, D.S.; Tran, H.N.; Woo, S.H. Effect of bentonite–macroalgae co-pyrolysis on biochar properties. J. Taiwan Inst. Chem. Eng. 2019, 104, 106–113. https://doi.org/10.1016/j.jtice.2019.08.017.
103. Rutkowski, P. Characteristics of bio-oil from catalytic pyrolysis of beverage carton waste. J. Anal. Appl. Pyrolysis 2013, 104, 609–617. https://doi.org/10.1016/j.jaap.2013.05.006.
104. Ellison, C.R.; Boldor, D. Mild upgrading of biomass pyrolysis vapors over iron–montmorillonite. Fuel 2021, 291, 120226. https://doi.org/10.1016/j.fuel.2021.120226.
105. Cai, W.; Zhu, X.; Kumar, R.; Zhu, Z.; Ye, J.; Zhao, J. Catalytic pyrolysis using montmorillonite-supported iron nanoparticles. Green Energy Resour. 2024, 2, 100085. https://doi.org/10.1016/j.gerr.2024.100085.
106. Ariöz, E. Catalytic fast pyrolysis of safflower biomass. Int. J. Energy Clean Environ. 2022, 23, 53–62.
107. Hong, W.; Zhang, Y.; Song, J.; Liu, H.; Zhang, L.; Wang, H.; Jiang, H. Phenol-enriched bio-oil using cobalt-modified montmorillonite. J. Anal. Appl. Pyrolysis 2023, 170, 105895. https://doi.org/10.1016/j.jaap.2023.105895.

108. Adjaye, J.D.; Bakhshi, N.N.; Katikaneni, S.P.R. Catalytic conversion of canola oil to fuels. Can. J. Chem. Eng. 1995, 73, 484–497. https://doi.org/10.1002/cjce.5450730408.
109. Fatimah, I.; Hidayat, A.; Setiawan, K.H. Aluminium pillared clay for bio-oil cracking. Asian J. Chem. 2010, 22, 7271–7280.
110. Budsaereechai, S.; Hunt, A.J.; Ngernyen, Y. Catalytic pyrolysis of plastic waste. RSC Adv. 2019, 9, 5844–5857. https://doi.org/10.1039/C8RA10058F.
111. Cai, W.; Kumar, R.; Zheng, Y.; Zhu, Z.; Wong, J.W.C.; Zhao, J. Clay catalysts for mixed plastic pyrolysis. Heliyon 2023, 9, e23140. https://doi.org/10.1016/j.heliyon.2023.e23140.
112. Li, K.; Lei, J.; Yuan, G.; Weerachanchai, P.; Wang, J.-Y.; Zhao, J.; Yang, Y. Pillared clays for catalytic pyrolysis of plastics. Chem. Eng. J. 2017, 317, 800–809. https://doi.org/10.1016/j.cej.2017.02.113.
113. Lei, J.; Yuan, G.; Weerachanchai, P.; Lee, S.W.; Li, K.; Wang, J.-Y.; Yang, Y. Thermal dechlorination and catalytic pyrolysis of plastic waste. J. Mater. Cycles Waste Manag. 2018, 20, 137–146. https://doi.org/10.1007/s10163-016-0555-3.
114. Li, K.; Wang, Y.; Zhou, W.; Cui, T.; Yang, J.; Sun, Z.; Min, Y.; Lee, J.-M. Catalytic pyrolysis over Co/Ni pillared montmoril-lonites. Chemosphere 2022, 299, 134440. https://doi.org/10.1016/j.chemosphere.2022.134440.
115. Jha, A.; Garade, A.C.; Shirai, M.; Rode, C.V. Metal-exchanged montmorillonite catalysts. Appl. Clay Sci. 2013, 74, 141–146. https://doi.org/10.1016/j.clay.2012.10.005.
116. Xu, H.; Wang, K.; Zhang, H.; Hao, L.; Xu, J.; Liu, Z. Ionic-liquid-modified montmorillonite-supported Ru catalysts. Catal. Sci. Technol. 2014, 4, 2658–2666. https://doi.org/10.1039/C4CY00250D.
117. Hengne, A.M.; Kadu, B.S.; Biradar, N.S.; Chikate, R.C.; Rode, C.V. Transfer hydrogenation of levulinic acid. RSC Adv. 2016, 6, 59753–59761. https://doi.org/10.1039/C6RA08637C.
118. Kasar, G.B.; Medhekar, R.S.; Bhosale, P.N.; Rode, C.V. Kinetics of levulinic acid hydrogenation. Ind. Eng. Chem. Res. 2019, 58, 19803–19817. https://doi.org/10.1021/acs.iecr.9b03748.
119. Wang, X.; Li, C.; Guo, X.; Wang, Z.; Cheng, R.; Xu, T.; Li, Y.; Wang, J.; Xu, H. Pd nanoparticles stabilized by modified montmorillonite. Front. Chem. 2022, 10, 961814. https://doi.org/10.3389/fchem.2022.961814.
120. Kumar, A.; Bal, R.; Srivastava, R. Ni-supported montmorillonite for hydrodeoxygenation of vanillin. ChemCatChem 2024, 16, e202301636. https://doi.org/10.1002/cctc.202301636.
121. Adilina, I.B.; Rinaldi, N.; Simanungkalit, S.P.; Aulia, F.; Oemry, F.; Stenning, G.B.G.; Silverwood, I.P.; Parker, S.F. Hydro-deoxygenation of guaiacol over pillared clay catalysts. J. Phys. Chem. C 2019, 123, 21429–21439. https://doi.org/10.1021/acs.jpcc.9b01890.
122. Kumar, M.; Upadhyay, S.N.; Mishra, P.K. Effect of montmorillonite on paper mill waste pyrolysis. Bioresour. Technol. 2020, 307, 123161. https://doi.org/10.1016/j.biortech.2020.123161 .
123. Wang, D.; Li, D.; Lv, D.; Liu, Y. Reduction of phenolic compounds via catalytic pyrolysis. BioResources 2014, 9, 4014–4021. https://doi.org/10.15376/biores.9.3.4014-4021.
124. Elfadly, A.M.; Zeid, I.F.; Yehia, F.Z.; Abouelela, M.M.; Rabie, A.M. Aromatic hydrocarbons from lignin catalytic pyrolysis. Fuel Process. Technol. 2017, 163, 1–7. https://doi.org/10.1016/j.fuproc.2017.03.033.

125. Adekayero, G.E.; Osial, M.; Oluwasina, O.O.; Omoruyi, O.E.; Olusegun, S.J.; Lajide, L. Clay-based catalyst for polypropylene pyrolysis. Mater. Chem. Phys. 2025, 343, 131030. https://doi.org/10.1016/j.matchemphys.2025.131030.
126. Chen, H.; Shan, R.; Li, S.; Zhao, F.; Zhang, Y.; Yuan, H.; Chen, Y. Clay minerals for controlling nitrogen pollutants in sludge pyrolysis. Sci. Total Environ. 2024, 913, 169535. https://doi.org/10.1016/j.scitotenv.2023.169535.
127. Jia, H.; Zhao, S.; Zhou, X.; Qu, C.; Fan, D.; Wang, C. Low-temperature pyrolysis of oily sludge using pillared bentonites. Arch. Environ. Prot. 2017, 43, 82–90. https://doi.org/10.1515/aep-2017-0027.
128. Mancini, I.; Dosi, F.; Defant, A.; Crea, F.; Miotello, A. Cellulose catalytic pyrolysis and bio-oil analysis. J. Anal. Appl. Pyrolysis 2014, 110, 285–290. https://doi.org/10.1016/j.jaap.2014.09.014.
129. Rutkowski, P. Pyrolytic behavior of cellulose in the presence of montmorillonite K10. J. Anal. Appl. Pyrolysis 2012, 98, 115–122. https://doi.org/10.1016/j.jaap.2012.07.012.
